# Characterization of Ikaria Heather Honey by Untargeted Ultrahigh-Performance Liquid Chromatography-High Resolution Mass Spectrometry Metabolomics and Melissopalynological Analysis

**DOI:** 10.3389/fchem.2022.924881

**Published:** 2022-07-22

**Authors:** Konstantinos M. Kasiotis, Eirini Baira, Styliani Iosifidou, Kyriaki Bergele, Electra Manea-Karga, Ioannis Theologidis, Theodora Barmpouni, Despina Tsipi, Kyriaki Machera

**Affiliations:** ^1^ Laboratory of Pesticides’ Toxicology, Benaki Phytopathological Institute, Athens, Greece; ^2^ General Chemical State Laboratory, Independent Public Revenue Authority (A.A.D.E), Athens, Greece

**Keywords:** mediterranean honey, pollen, ikaria honey, metabolomics, UHPLC-HRMS, orbitrap, melissopalynology, chemometrics

## Abstract

Honey represents a valuable food commodity, known since ancient times for its delicate taste and health benefits due to its specific compositional characteristics, mainly the phenolic compound content. “Anama” honey is a monofloral honey produced from the nectar of *Erica manipuliflora* plant, a heather bush of the Greek island of Ikaria, one of the Mediterranean’s longevity regions. “Anama” is characterized by a unique aroma and taste, with a growing demand for consumption and the potential to be included in the list of products with a protected designation of origin. The aim of this study was to determine the chemical and botanical profile of authentic Anama honey samples and find similarities and differences with honey samples of a different botanical origin from the same geographical area. Untargeted Ultrahigh-Performance Liquid Chromatography-Hybrid Quadrupole-Orbitrap High-Resolution Mass Spectrometry (UHPLC-HRMS) metabolomics study was conducted on authentic heather, pine, and thyme honey samples from Ikaria and neighboring islands. The Principal Component Analysis (PCA), Orthogonal Projections to Latent Structures Discriminant Analysis (OPLS-DA), and differential analysis were performed using the entire metabolic profile of the samples and allowed the identification of chemical markers for sample discrimination. Thirty-two characteristic secondary metabolites (cinnamic acids, phenolic acids, flavonoids, terpenes) and other bioactive phenolic compounds, some of them not previously reported in a heather honey (aucubin, catalpol, domesticoside, leonuriside A, picein among others), emerged as potential chemical indicators of Anama honey. Melissopalynological analysis was also carried out to decipher the botanical and geographical origin of Anama honey. The relative frequency of the pollen of dominant plants of the Ericaceae family and a multitude of nectariferous and nectarless plants contributing to the botanical profile of Anama was evaluated. The identification of the pollen sources enabled a potential correlation of differentially increased secondary metabolites and chemicals with their botanical origin. The physicochemical profile of Anama was also determined, including the parameters of pH, color, electrical conductivity, diastase, moisture, as well as sugars, supporting the high quality of this heather honey.

## 1 Introduction

Honey is a natural product acclaimed since ancient times both for its taste and its various beneficial effects on human health (Hills et al., 2019; Kamaruzzaman et al., 2019; Nguyen et al., 2019). Though not consumed in large quantities (the average consumption of honey per capita and per day in Europe is less than 5 g/capita/day) (SANTE/11956/2016, 2018), honey and other apiculture products (e.g., royal jelly) are still an important source of valuable nutrients for humans, as they are extremely rich in bioactive components. The main components of honey are fructose and glucose, and also many other disaccharides, such as maltose and sucrose. Honey also contains small amounts of vitamins and minerals, as well as organic acids, amino acids, proteins, enzymes, and polyphenols, mainly flavonoids, and phenolic acids. The chemical composition of honey and its content of bioactive compounds is expected to vary to a great extent depending on the floral nectar that bees forage on, which, in turn, is influenced by the plant species and geographical origin. Phenolic compounds responsible for antioxidant and anti-inflammatory capacity of honey are secondary metabolites synthesized by plants and could be used as botanical markers for classification and authentication, especially in the case of monofloral honeys (Ciulu et al., 2016). Monofloral (or unifloral) honeys are considered those that come wholly or mainly from the indicated plant source and possess the organoleptic, physicochemical, and microscopic characteristics of the source (Council-Directive, 2001; Oddo et al., 2004). They must also conform to existing legislative criteria (Thrasyvoulou et al., 2018). Some monofloral honeys are more appreciated than others due either to their flavor and aroma properties or to their pharmacological attributes. The most famous monofloral honey used for medical purposes is Manuka honey from New Zealand, but several studies have revealed that other monofloral honeys, such as heather honey, are also very effective (Salonen et al., 2017; Gourdomichali and Papakonstantinou, 2018). Heather honey, obtained from plants that belong to Ericaceae family, is relatively rare and very appreciated by consumers, due to its sensorial properties and potential health benefits (Cianciosi et al., 2018; Rodríguez-Flores et al., 2021). Greece is a Mediterranean country with a high production of heather honey. Several different species of Erica plants contribute to the production of this honey type, especially *Erica arborea* and *E. manipuliflora*, among others. In the last 5 years, the monofloral heather honey from *Erica manipuliflora* plant of Ikaria (and Fournoi) island, one of the Mediterranean’s longevity regions, called “Anama” (Greek, Άναμα), is at the forefront of the Greek apiculture and honey community due to its unique flavor and organoleptic features. It has an intense aroma and its taste is pungent, smoky, slightly bitter, and mildly sweet, which leaves a long aftertaste. It is on the list of three products from the North Aegean prefecture to be assessed for products of protected designation of origin and the relevant registration file will be prepared by the Directorate of Rural Development of the North Aegean Region (Anama-honey, 2021).

The current Regulation (EU) 625/2017 on official food control prioritizes the control of food authenticity (REG-625/2017, 2017), including protected designations of origin, geographical indications, and traditional specialties (Council-Directive, 2001). A wide range of analytical methods has been developed for food characterization, quality, and authenticity testing; yet, in the last few years, novel methods principally based on the advances in mass spectrometry (MS) techniques are applied (Cook and Nightingale, 2018; Braconi et al., 2021). These omics-based methods are moving toward the maximum collection of information (chemical context, markers) on sample composition, fostering substantially this domain with proven application in honey commodities (Koulis et al., 2021). Complementary to this, and for honey, in particular, one of the most important analyses is the melissopalynological (pollen) analysis, which comprises the study of the whole range of plants that bees visit, by identifying the pollen deposited in the honey and extracting information about the botanical and geographical origin of the honey. Pollen analysis can also provide evidence of the presence of exogenous elements in honey, such as those that can result from bee feed, sometimes of dubious quality (von der Ohe et al., 2004). In the same direction, metabolomic approaches in combination with robust statistical analysis are pivotal tools in distinguishing diverse types of honey or investigating their taste and maturation attributes, disclosing characteristic-unique chemicals for the discrete categories (Guo, 2021; Koulis et al., 2021; Montoro et al., 2021).

To our knowledge, the already published data regarding the Greek heather honey chemical profile is very limited. More specifically, a recently published work has incorporated Ultrahigh Performance Liquid Chromatography High-Resolution Mass Spectrometry (UHPLC-HRMS) targeted and untargeted metabolomics analysis to discriminate between honey samples of different botanical sources from Greece and Poland. However, the exact geographical origin of the Greek samples was not provided, while melissopalynological and physicochemical analyses were not conducted (Koulis et al., 2021). Another work performed by using solid-phase microextraction and gas chromatography–mass spectrometry has been focused on volatile constituents of Greek heather honey (Xagoraris et al., 2021).

In this study, heather honey samples from Ikaria Island were investigated through an untargeted UHPLC-HRMS metabolomics study to elucidate the profile of secondary metabolites and chemicals. Using advanced chemometrics tools, the results were compared to the metabolomic results of pine and thyme honey from the same geographical origin area collected at different times of the year, to unveil potential chemical markers of this unique type of Anama honey. Extensive melissopalynological analysis performed on all honey samples, revealed a diversity of plants contributing to Anama honey composition when compared to the other Aegean monofloral pine and thyme honeys. For the first time, the identification of the pollen sources enabled a potential correlation of differentially increased secondary metabolites and chemicals with their botanical sources. Physicochemical parameters including the determination of pH, color, electrical conductivity, diastase, moisture, as well as sugars, were also performed, supporting the high quality of heather Anama honey.

## 2 Materials and Methods

### 2.1 Honey Samples

Authentic honey samples (n = 50) were provided by the beekeeping and rural cooperatives of five Aegean islands for consecutive years 2018, 2019, and 2020. Samples were collected immediately after harvesting and kept at -18°C until analysis. Harvesting was conducted in three different periods of the year, according to the botanical origin of honey.

Anama honey samples (n = 10) were collected from the islands of Ikaria and Fournoi between November and January 2018–2019 and 2019–2020. These islands were considered as one group due to their vicinity, and transferring of hives from one island to the other by the beekeepers. Thyme honey samples (n = 10) were also collected from Fournoi (June to August of 2018, 2019, and 2020), accompanied by the same number of samples from Kea (n = 10) and Syros (n = 10) islands. Pine honey samples (n = 10) were collected from Samos Island (August 2018–2020), the closest island to Ikaria and Fournoi islands.

### 2.2 Chemicals and Reagents

Acetonitrile (ACN), methanol (MeOH), and formic acid of liquid chromatography–mass spectrometry (LC-MS) grade were obtained from Merck (Darmstadt, Germany). Ultra-pure water (H_2_O) was produced from SG Millipore apparatus. Abscisic acid was obtained from Sigma Aldrich (Seelze, Germany). Discovery^®^ octadecylsilane (DSC-C_18_) solid-phase extraction (SPE) cartridges (500 mg, 6 ml) were purchased from Sigma Aldrich (Supelco, Bellefonte, PA, United States). Nylon filters (0.22 μm) were obtained from Macherey-Nagel (Duren, Germany). All reagents and chemicals were of analytical grade. (+)-Abscisic acid analytical standard (≥ 98%) was obtained from Sigma Aldrich (Seelze, Germany).

### 2.3 Sample Preparation for UHPLC-HRMS

Sample preparation was adapted from a published procedure (Di Marco et al., 2018). Briefly, honey (3 g) was homogenized with the aid of a glass rod in 7 ml of acidified ultrapure H_2_O (pH = 2.2). Then, the mixture was loaded on a pre-activated DSC-C_18_ SPE cartridge [activation was performed by succeeding elution of MeOH (3 ml) and H_2_O (3 ml)]. Consequently, the SPE cartridge was washed with acidified H_2_O (2 ml) and 5 ml of ultrapure H_2_O. Compounds of interest were eluted with 3.5 ml of a MeOH/ACN (2:1, v/v) solution. Then, the eluate was evaporated to dryness using a rotary evaporator (bath temperature not exceeding 30°C) and reconstituted in MeOH/H_2_O (7:3, v/v). After filtration with Nylon filters (0.22 μm), the extract was injected into UHPLC-HRMS system.

### 2.4 Ultrahigh Performance Liquid Chromatography Orbitrap High-Resolution Mass Spectrometry Analysis of Honey Extracts

Samples were analyzed with a Dionex Ultimate 3000 UHPLC system (Thermo Fisher Scientific, San Jose, CA, United States) linked to a Q-Exactive Orbitrap HRMS (Thermo Fisher Scientific, San Jose, CA, United States). Chromatographic separation was performed on a Hypersil Gold UPLC C18 (2.1 mm × 150 mm, 1.9 μm) reversed phased column (Thermo Fisher Scientific, San Jose, CA, United States). The ionization was performed using heated electrospray (ESI) in both positive and negative modes. Chromatographic and mass spectrometry conditions and parameters are described in the previous work of our group (Stavropoulou et al., 2021). To monitor the stability and repeatability of the instrumental procedure, a quality control (QC) sample was analyzed three times before and at the end of the sequence, and after every six experimental samples. The QC sample was prepared as a pool of all study samples.

### 2.5 Data Processing and Multivariate Statistical Analysis

Data were processed with Compound Discoverer 2.1 (Thermo Fisher Scientific, San Jose, CA, United States). The workflow was an untargeted metabolomics approach for detecting unknown compounds and searching in online databases. The software was used for peak detection, deconvolution, deisotoping, alignment, gap-filling, and composition prediction procedures. Peaks in which the QC sample coverage was less than 50% and the relative standard deviation of the areas under the peaks was more than 30% were excluded. The generated peak lists of accurate masses and retention times paired with corresponding intensities for all detected peaks were exported as a .csv file, imported to Microsoft Excel 2010, and manipulated appropriately using the “concatenate,” “round” and “transpose” commands. The R package *MetaboDiff* (Mock et al., 2018) was implemented for the differential analysis of metabolomic data and for the calculation of Log2 Fold Changes and adjusted *p*-values (Benjamini-Hochberg).

The MS data were then subjected to multivariate statistical analysis (MVA) i.e., Principal Component Analysis (PCA) and Orthogonal Projections to Latent Structures Discriminant Analysis (OPLS-DA) using the *ropls* R Bioconductor package (Thévenot et al., 2015) to determine the optimal number of components, confirm the validity of the model by permutation testing, detect outliers and perform feature selection with Variable Importance in Projection (VIP) scoring from OPLS-DA models. Variables that exhibited a VIP score greater than 1.5, combined with an adjusted *p*-value ≤0.05 and Log2 Fold Change (Anama *vs*. Thyme or Anama *vs*. Pine) >1.5 from differential analysis, were considered as contributing the most to group separation. The common differentially increased variables on Anama honey compared to thyme and pine honey were forwarded to putatively annotation. Databases such as mzCloud (https://www.mzcloud.org/), MassBank (www.massbank.jp), and Metlin (http://metlin.scripps.edu) were used for putative annotation by applying *m/z* tolerance of 5 ppm and taking into consideration the isotope distribution similarity and MS/MS fragmentation pattern deriving from the Data Dependent Acquisition (DDA) methodology. For the compounds for which there was no record in the aforementioned databases *in silico* fragmentation, the approach was applied using the MetFrag online tool, using 5 ppm search tolerance and 0.001 mass deviations to match generated fragments against MS/MS peaks (Wolf et al., 2010). For the MetFrag workflow, the candidate structures were retrieved from the databases PubChem (PubChem, n.d.) and Kegg. For the selection of the compounds >0.8 final scores were applied. The score for every matching is presented in Supplementary Table S1.

### 2.6 Melissopalynological Analysis

For the Melissopalynological analysis, the methods of Louveaux & von der Ohe were applied (von der Ohe et al., 2004; Louveaux et al., 1978). In this respect, a solution of 10 g of honey in 20 ml of distilled water is centrifuged for 10 min at 1,000 *g*. After decanting the supernatant liquid, the sediment is suspended again in 20 ml of distilled water and centrifuged for 5 min at 1,000 *g*. The supernatant liquid is decanted, and the sediment is spread over a microscope slide on a heating plate (40°C). The sediment is left to dry and then is colored with some drops of a solution of fuchsine (∼0.05‰ w/v in ethanol/water 1/1). After drying (40°C), a coverslip with a drop of glycerin jelly (mounting medium; Kaiser’s Glycerol Gelatin TM Merck 1.09242.0100) is placed upon the sediment and the slide is left on the heating plate for another 5 min. The microscope slide is investigated under the microscope (400x). For the determination of relative frequencies of pollen types, 500 to 1000 pollen grains are counted (Behm et al., 1996). To detect all possible plant sources, especially those that exhibit very large pollen grains, which are not expected to be present in a substantial amount, the entire slide was scanned at 50x magnification. Quantitative melissopalynological analysis was achieved using Slide Grids, 20 mm × 20 mm. At least 10 mm^2^ were counted.

### 2.7 Physicochemical Characteristics

The determination of physicochemical parameters of Anama honey samples was achieved by applying the “Harmonised Methods of the International Honey Commission (IHC)” (IHC, 2009). Specifically, the following protocols of IHC methods were applied: The refractive index (RI) of honey was measured using a refractometer. The water content was determined from the refractive index of honey by reference to a standard table. The electrical conductivity of a solution of 20 g dry matter of honey in 100 ml distilled water was measured using an electrical conductivity cell, at 20°C. The pH of a solution of 10 g honey in 75 ml of carbon dioxide-free water was measured using a pH meter, accurate to 0.01 units. The determination of the hydroxymethylfurfural (HMF) content was based on the measurement of UV absorbance of HMF at 284 nm (after White). In order to avoid the interference of other components at this wavelength, the difference between the absorbances of a clear aqueous honey solution and the same solution after the addition of bisulfite was determined. The HMF content was calculated after the subtraction of the background absorbance at 336 nm. The determination of the diastase activity of honey was performed by a photometric method in which an insoluble blue-dyed cross-linked type of starch was used as the substrate (Phadebas tablets). This was hydrolyzed by the enzyme, yielding blue water-soluble fragments, determined photometrically at 620 nm. The absorbance of the solution is directly proportional to the diastase activity of the sample. The determination of sugars was performed in a filtrated solution of 10 g of honey in 100 ml of water by High-Pressure Liquid Chromatography (HPLC) using a RI-detector. Peaks were identified on the basis of their retention times. Quantitation was performed according to the external standard method on peak areas or peak heights.

## 3 Results

### 3.1 UHPLC-HRMS Data Analysis and Chemometrics

Application of the untargeted LC-MS screening in the three different honey types, as described in previous work (Stavropoulou et al., 2021), resulted in a peak list containing the accurate mass *vs.* intensity of the detected features. Then, an untargeted metabolomics workflow encompassing statistical analysis was performed to identify the most important markers that discriminate Anama from pine and thyme honey samples.

**FIGURE 1:**
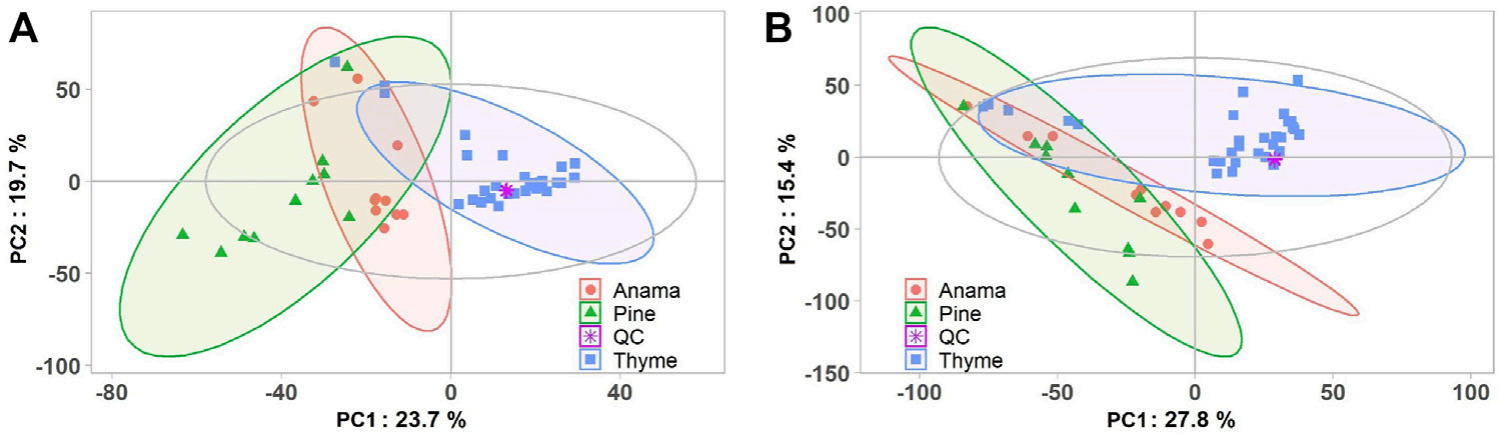
PCA score plots **(A,B)** of the honey Anama (orange), pine (green), thyme (blue) and QC (purple) samples after UHPLC-HRMS analyses in negative (R2X = 0.527) **(A)** and positive (R2X = 0.524) **(B)** ion mode, respectively.

**FIGURE 2:**
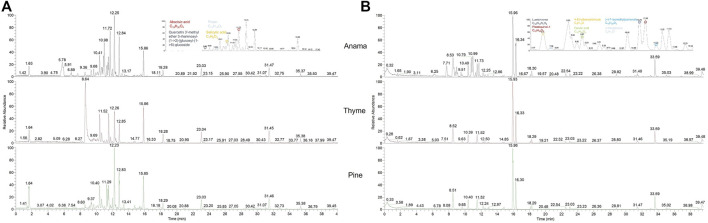
UHPLC-HRMS representative chromatograms of Anama, thyme, and pine (from up to bottom respectively) honey extracts in negative [**(A)**, ESI-] and positive [**(B)**, ESI+] ionization mode.

More specifically, aiming to identify potential chemical markers of Anama honey when paralleled with pine and thyme honey, multivariate statistical analysis (MVA) i.e., Principal Component Analysis (PCA), Orthogonal Projections to Latent Structures Discriminant Analysis (OPLS-DA) and differential analysis were performed using the entire metabolic profile of the samples (all features obtained). QC clustering in PCA analysis in both modes confirmed the reliability of the system and the quality of the acquired data. Based on the results of PCA analysis, a tendency of separation of the three different honey types could be observed in both modes (Figure 1, PCA and OPLS-DA loading plots are presented in Supplementary Figures S1–S6). These findings indicate that the majority of their chemical content was different for the three honey types, whereas some metabolites were similar. Two representative chromatograms of the three types of honey are presented in Figure 2.

**FIGURE 3:**
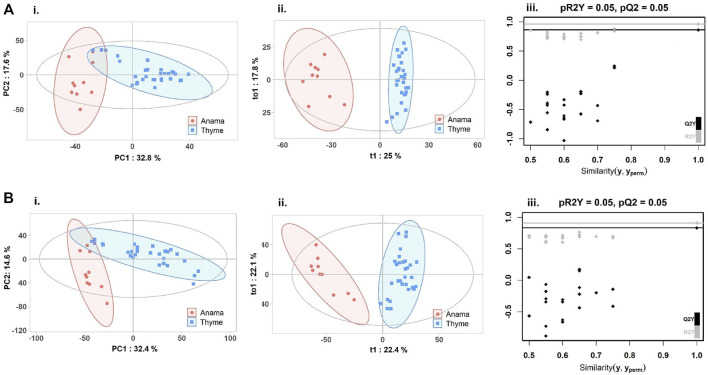
PCA **(Ai, Bi)**, OPLS-DA **(Aii, Bii)** score plots and permutation tests **(Aiii, Biii)** after the comparison of Anama (orange) and Thyme (blue) honeys employing UHPLC-HRMS analysis in negative **(A)** and positive **(B)** ion mode, respectively. The goodness of fit and prediction of the models were for PCA analysis **(Ai)** R2X = 0.504, **(Bi)** R2X = 0.586 and for OPLS-DA analysis **(Aii)** R2X = 0.591, R2Y = 0.963, Q2 = 0.862, **(Bii)** R2X = 0.52, R2Y = 0.909, Q2 = 0.831, *x* and *y* axes of panels (ii) correspond to T-score (t1) and Orthogonal T-score (to1) respectively.

**FIGURE 4:**
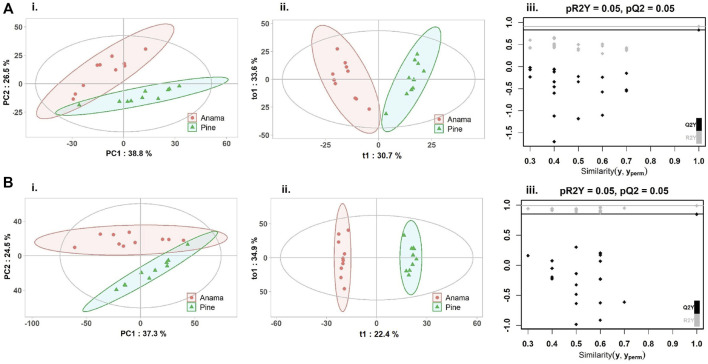
PCA **(Ai, Bi)**, OPLS-DA **(Aii, Bii)** score plots and permutation tests **(Aiii, Biii)** after the comparison of Anama (orange) and pine (green) honeys employing UHPLC-HRMS analysis in negative **(A)** and positive **(B)** ion mode, respectively. The goodness of fit and prediction of the models were for PCA analysis **(Ai)** R2X = 0.653, **(Bi)** R2X = 0.618 and for OPLS-DA analysis **(Aii)** R2X = 0.644, R2Y = 0.908, Q2 = 0.828, **(Bii)** R2X = 0.707, R2Y = 0.991, Q2 = 0.849, *x* and *y* axes of panels (ii) correspond to T-score (t1) and Orthogonal T-score (to1) respectively.

To further verify the differences in metabolic profiles, OPLS-DA was carried out which showed a clear separation between Anama and other monofloral honeys (Figures 3, 4). Features with Variable Importance in Projection (VIP) scoring from OPLS-DA models greater than 1.5 combined with adjusted *p*-value ≤0.05 and Log2 Fold Change (Anama *vs*. Thyme and Anama *vs*. Pine) >1.5 from differential analysis, were considered as contributing the most to group separation.

**FIGURE 5:**
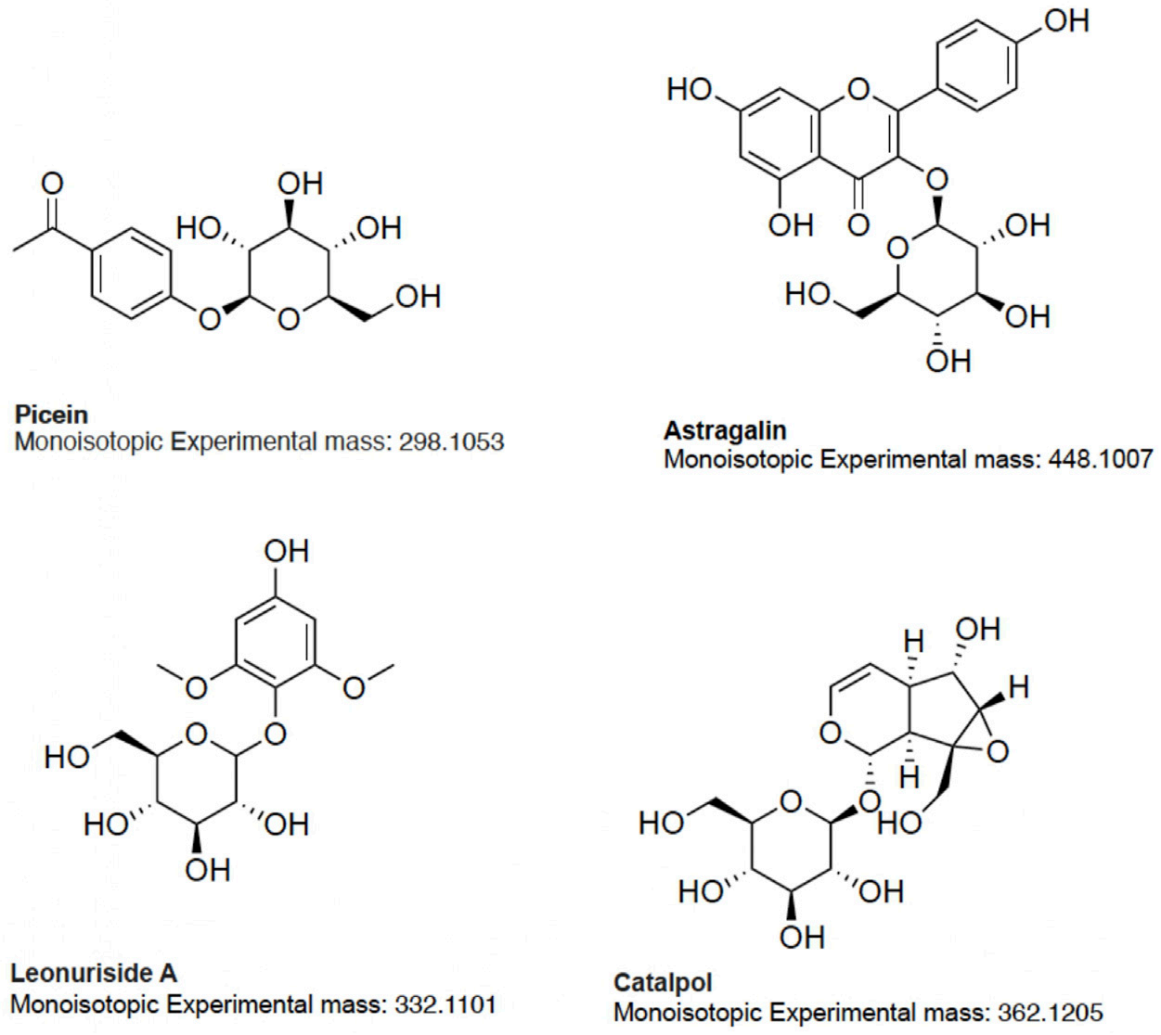
Chemical structures of selected differentially increased compounds in Anama honey.

**FIGURE 6:**
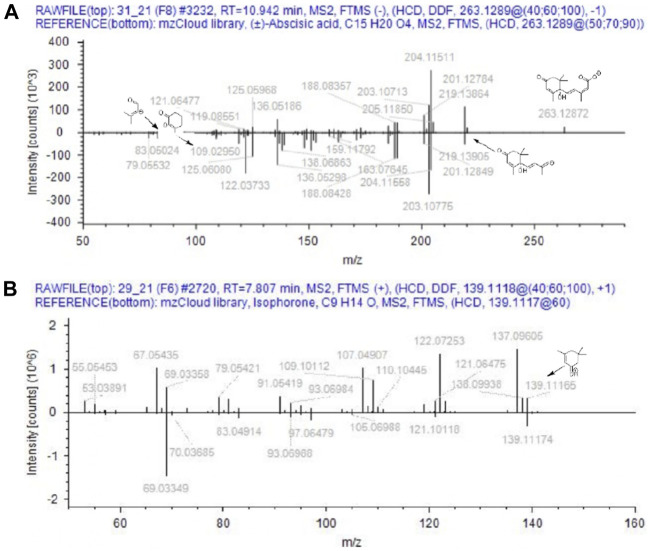
Comparison of MS/MS data from Anama honey (top spectra) to MS/MS of (±)-abscisic acid **(A)** and isophorone **(B)** of mzCloud database (bottom spectra).

Based on the aforementioned statistical criteria, 32 characteristic compounds were found to be increased in all Anama samples analyzed, when compared to pine and to thyme honeys (using the obtained peak area for relative quantification). Similarly, according to the statistical analysis and the log2FC values, various compounds were found to be differentially increased in thyme and pine honey compared to Anama. Some key bioactive molecules putatively annotated in Anama honey that can serve as specific chemical markers of Anama honey, including their experimental monoisotopic mass, retention time (tR), MS/MS fragment ions, and molecular formula, are presented in Table 1. For the compounds that the putative annotation was based on *in silico* fragmentation, the final score is presented in Supplementary Table S1. Figure 5 presents some representative chemical structures of characteristics markers in Anama honey. (±)-Abscisic acid (Table 1) was identified after a comparison of MS/MS data with data produced from mzCloud database (Figure 6) and authentic analytical standard solution injection.

**TABLE 1:** Monoisotopic mass and MS/MS fragment ions of selected secondary metabolites and compounds, potential chemical markers of Anama honey.

Compound annotation	Class	Monoisotopic Mass (Da) experimental	tR (min)	MS/MS fragment ions (*m/z*)	Molecular formula	Adduct ion	Previous occurrence in heather honey^a^	References^b^
(±)-Abscisic acid^e^	Terpene	264.1361	11.74	204.11/219.13/203.10	C_15_H_20_O_4_	[M-H]^−^	Y	Leu et al. (2012)
Acetophenone^e^	Acetophenones	120.0575	6.22	103.05/91.05	C_8_H_8_O	[M+H]^+^	Y	(Yang et al., 2012; Stefanikova et al., 2021)
2-Anisic acid^e^	Carboxylic Acid	152.0474	10.81	109.06/94.04/135.04	C_8_H_8_O_3_	[M+H]^+^	N	(Sytar et al., 2018)^c^
Astragalin^e^	Flavonoid (kaempferol glucoside)	448.1007	10.51	255.03/227.04	C_21_H_20_O_11_	[M-H]^−^	Y	Goslinski et al. (2021)
Aucubin^f^	Terpene	346.1258	11.04	263.07/283.08	C_15_H_22_O_9_	[M+H]^+^	N	(Tundis et al., 2008; Li et al., 2020; Olajide et al., 2020; Potacnjak et al., 2020; Potocnjak et al., 2021)^d^
Catalpol^f^	Iridoid glycoside	362.1205	8.63	285.05/299.07	C_15_H_22_O_10_	[M+H]^+^	N	Zhang et al. (2021)
p-Coumaroyl-D-glucose^f^	Glucose derivative	326.1003	11.23	121.06/135.04	C_15_H_18_O_8_	[M+H]^+^	N	Tsagkarakou et al. (2021)
Cuminaldehyde^e^	Benzaldehyde derivative	148.0889	16.43	149.09/79.05	C_10_H_12_O	[M+H]^+^	N	Anand et al. (2019)
D-tryptophan^e^	Amino acid	204.0899	10.81	91.05/115.05/117.05	C_11_H_12_N_2_O_2_	[M+H]^+^	Y	Kowalski et al. (2017)
Dehypoxanthine	Inosine derivative	296.0897	7.08	135.04/153.05	C_14_H_16_O_7_	[M+H]^+^	N	Manion-Sommerhalter et al. (2021)
Futalosine^f^								
3,4-Dimethoxycinnamic acid^e^	Hydroxycinnamic acids	208.0734	11.02	91.05/103.05/79.5	C_11_H_12_O_4_	[M+H]^+^	N	Margaoan et al. (2021)
Domesticoside^f^	Phenolic glycoside	344.1109	8.88	241.07/149.02	C_15_H_20_O_9_	[M-H]^-^	N	Dawood et al. (2020)
4-Ethylbenzaldehyde^e^	Aldehyde	134.0731	7.73	79.05/95.05	C_9_H_10_O	[M+H]^+^	N	—
2-Ethylphenol^f^	Phenol	122.0732	7.98	67.05/79.05	C_8_H_10_O	[M+H]^+^	N	
Ferulic acid^e^	Hydroxycinnamic acid	194.0580	8.90	177.05	C_10_H_10_O_4_	[M+H]^+^	Y	Paiva et al. (2013)
Ganolucidic acid B^f^	Terpene	502.3296	17.73	119.08/173.13/203.14	C_30_H_46_O_6_	[M+H]^+^	N	(Kikuchi et al., 1986; Yang Y. et al., 2019)
5-Hydroxyferulic acid methyl ester^f^	Carboxylic acid	210.0524	11.21	151.03/136.01	C_10_H_10_O_5_	[M-H]^-^	N	Paiva et al. (2013)
(+)-7-Isomethyljasmonate^f^	Jasmonate derivative	224.1412	11.35	123.11/85.02	C_13_H_20_O_3_	[M+H]^+^	N	Heitz et al. (2016)
Isophorone^e^	Norisoprenoid	138.1045	7.74	69.03/139.11	C_9_H_14_O	[M+H]^+^	Y	Lamari et al. (2018)
Kaempferol-3-O-galactoside (or trifolin)^f^	Flavonoids	448.1007	11.05	271.02/300.02/314.04	C_21_H_20_O_11_	[M-H]^-^	N	Kim et al. (2016)
Leonuriside A^f^	Phenolic glycoside	332.1101	8.69	165.05/69.03/109.02	C_14_H_20_O_9_	[M+H]^+^	N	(Thai et al., 2017; Nguyen et al., 2018)
Lumichrome^e^	Flavin	242.0802	10.81	172.01/216.01	C_12_H_10_N_4_O_2_	[M+H]+	Y	Chantarawong et al. (2019)
6-Methoxyluteolin 7-rhamnoside^f^	Flavonoid	462.1163	11.54	243.03/271.02/314.04	C_22_H_22_O_11_	[M-H]^−^	N	do Nascimento et al. (2019)
Methyl-3-aminobenzoate^e^	Aromatic carboxylic acid derivative	151.0634	6.67	152.07/135.04	C_8_H_9_NO_2_	[M+H]^+^	N	Kronenberger et al. (2020)
Phenyllactic acid^f^	Organic acid	166.0633	8.48	121.06/149.05	C_9_H_10_O_3_	[M+H]^+^	Y	Chaudhari and Gokhale, (2016)
Picein^f^	Phenol	298.1053	10.35	125.05/57.03/85.02	C_14_H_18_O_7_	[M-H]^−^	N	Kesari et al. (2020)
Plastoquinol-1^f^	Quinone	206.1307	11.01	137.09/71.04/109.06	C_13_H_18_O_2_	[M+H]^+^	N	Borisova-Mubarakshina et al. (2019)
4-Propylphenol^f^	Phenol	136.0889	11.02	109.06/94.04/53.03	C_9_H_12_O	[M+H]^+^	N	Nikaido et al. (1981)
Quercetin 3′-methyl ether 3-rhamnosyl-(1->2)-[glucosyl-(1->6)-glucoside]^f^	Flavonoids	786.2227	9.45	299.02/314.04/271.02	C_34_H_42_O_21_	[M-H]^−^	N	—
Riboflavin^f^	Vitamin (isoalloxazine derivative)	376.1384	9.27	229.09/257.10/175.08	C_17_H_20_N_4_O_6_	[M+H]^+^	Y	Suwannasom et al. (2020)
Salicylic acid^e^	Phenolic acid	138.0306	10.80	137.02/93.03	C_7_H_6_O_3_	[M-H]^−^	N	Randjelović et al. (2015)
Trichocarpin^f^	Organic acid derivative	406.1277	12.4	240.06/272.05/197.05	C_20_H_22_O_9_	[M-H]^−^	N	Mattes et al. (1987)

a Yes/No, Y/N.

b Reference to occurrence and/or bioactivity.

c Reference to bioactivity of all isomers.

d Reference to the chemical class bioactivity.

e Compounds putatively annotated based on databases.

f Compounds putatively annotated based on in silico fragmentation.

### 3.2 Melissopalynological Analysis

**FIGURE 7:**
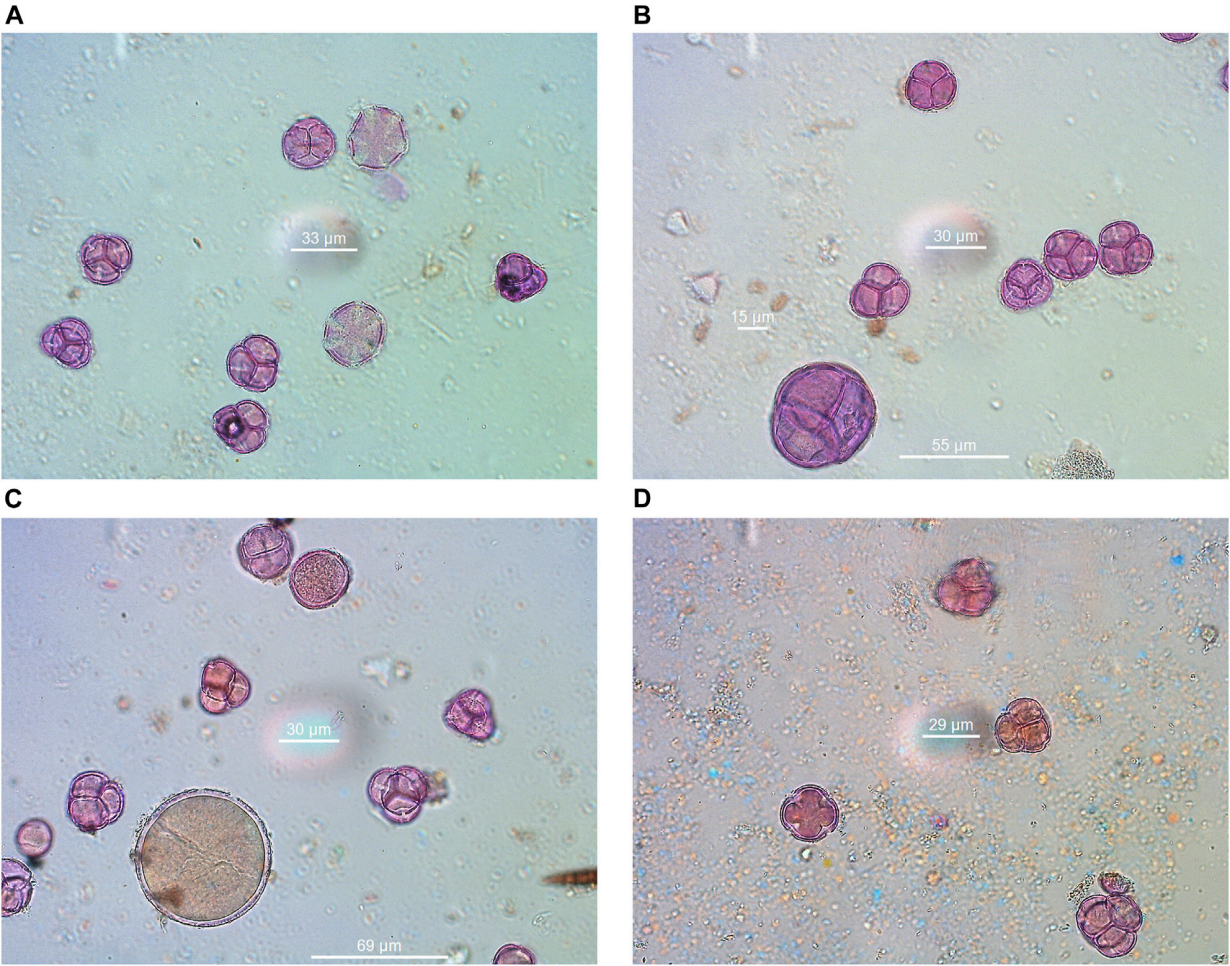
Indicative pollen grains were identified in Anama honey. **(A)** *Erica*-*Thymus*, **(B)** *Erica*-*Arbutus*-*Myrtus*, **(C)** *Erica*-*Crocus*-*Pistacia*, **(D)** *Erica*-*Ceratonia*.

The results of melissopalynological analysis showed that all Anama honeys were compliant with the requirements of the National legislation of Greece (Author Anonymous, 2022) as well as with the regulations of other European countries about heather honey (Thrasyvoulou et al., 2018), having a percentage of *Erica* sp. pollen above 45% (Table 2, Supplementary Table S4). National regulations are important due to the lack of International or European standards for monofloral honeys. Indicative photos with pollen grains are presented in Figures 7A–D. Although Erica pollen dominates (av. 85%, range 63–99%, Supplementary Table S4), a large number of other species were detected (av. 24 taxa per sample, range 15–39, Table S4), characteristic of the rich flora of the island. The relative proportion of the rest of the taxa in honey is low, a fact that is natural due to the overabundance of pollen grains from Erica. However, their presence is crucial for the identification of the geographical origin of honey, as it is a good fingerprint of the environment where the honey comes from (von der Ohe et al., 2004; Louveaux et al., 1978). Alongside, melissopalynological analysis did not detect any exogenous elements resulting from bee feed, e.g., starch granules, demonstrating the authenticity of honey.

**TABLE 2:** Pollen spectrum of honeys studied. Data presented as frequency classes.

Nectariferous plants		VF = very Frequent >45%			F = Frequent 16–45%			R = rare 3–16%		S =Sporadic <3%	
		Samples									
**Family**	**Genus**	**IKF18-23**	**IKF18- 24**	**IKA18- 29**	**IKF19- 22**	**IKF19- 23**	**IKA19-27**	**IKA19-28**	**IKA19-30**	**IKF20-13**	**IKA20-14**
Aizoaceae	*Carpobrotus*							S	S		
Apiaceae					S	S	S	S	S		S
Araliaceae	*Hedera*						S	S	S		R
Asparagaceae	*Asparagus*	S	S	S		S	S	S	S		S
Asteraceae	*Anthemis*					S			S		
Asteraceae	*Carthamus*				S						
Asteraceae	*Centaurea*				S			S	S	S	
Asteraceae	*Dittrichia/Inula*	S	S		S	R		S	S	S	
Asteraceae	*Onopordum*						S	S	S	S	S
Asteraceae	*Taraxacum*			S	S	S	S		S		S
Brassicaceae	*Brassicaceae (Sinapis)*			S				S			S
Cactaceae	*Opuntia*				S						
Caesalpiniaceae	*Ceratonia*	R	R	S		R	S	S	S	S	S
Campanulaceae	*Campanulaceae*				S				S		
Colchicaceae	*Colchicum*										S
Convolvulaceae	*Convolvulus*				S	S				S	S
Cucurbitaceae	*Cucurbita*								S		
Dipsacaceae	*Knautia*									S	
Ericaceae	*Arbutus*	S		S			S	S	S	S	S
Ericaceae	*Erica*	VF	VF	VF	VF	VF	VF	VF	VF	VF	VF
Fabaceae	*Acacia*								S		S
Fabaceae	*Anthyllis hermanniaeT*				S						
Fabaceae	*Trifolium*			S				S			
Fabaceae	*Vicia*										S
Fabaceae							S	S	S		
Fagaceae	*Castanea*										S
Hyacinthaceae	*Drimia*										S
Iridaceae	*Crocus*			S			S		S		S
Lamiaceae	*Ballota*					S					
Lamiaceae	*Lavandula*	S		S		S	S	S	S		S
Lamiaceae	*Ocinum*	S									
Lamiaceae	*Salvia*									S	
Lamiaceae	*Satureja*							S	S		
Lamiaceae	*Teucrium*				S	S					
Lamiaceae	*Thymbra/Thymus*	S	S		F	F		S	S	S	S
Lauraceae	*Laurus/Persea*								S		
Liliaceae	*Liliaceae*							S	S		
Malvaceae	*Malvaceae*										S
Myrtaceae	*Myrtus*						R	S	S		
Oxalidaceae	*Oxalis*			S			S	S	S		
Ranunculaceae	*Clematis*										S
Rosaceae	*Pyrus-Prunus*			S			S				
Styracaceae	*Styrax*			S			R	S	S		S
Zygophyllaceae	*Tribulus*	S									

Nectariferous plants		Samples									
**Family**	**Genus**	**IKF18-23**	**IKF18- 24**	**IKA18- 29**	**IKF19- 22**	**IKF19- 23**	**IKA19-27**	**IKA19-28**	**IKA19-30**	**IKF20-13**	**IKA20-14**
Anacardiaceae	*Pistacia*	S	S	S	S	S	R	S	S	S	R
Arecaceae/Palmae		S	S								
Betulaceae/Corylaceae									S		S
Caryophyllaceae							S		S		
Chenopodiaceae/Amaranthaceae								S			
Cistaceae	*Cistaceae*		S	S	S	S	F	R	R	S	R
Cupressaceae	*Cupressaceae*	S	S	S		S				S	
Fabaceae	*Calicotome-Genista*	S	S	S	R	F			S		
Fagaceae	*Quercus coccifera Τ*		S	R		S	R	R	R	S	F
Fagaceae	*Quercus ithaburensis Τ*							S	S		S
Juglandaceae	*Juglans*			S					S		S
Oleaceae	*Fraxinus*						S				
Oleaceae	*Olea*	S	S	S	F	R	R	S	S	S	S
Oleaceae	*Phillyrea*										S
Papaverceae	*Papaver*				S						
Pinaceae	*Pinaceae*		S	S		S	S	S	S		
Plantaginaceae	*Plantago*										S
Platanaceae	*Platanus*										S
Polygonaceae	*Rumex*		S		S				S		
Primulaceae	*Cyclamen*	S	S	S		S				R	S
Rafflesiaceae	*Cytinus*			S							
Ranunculaceae	*Ranunculus*					S		S			S
Rubiaceae	*Galium*						S				
Scrophulariaceae	*Verbascum*		S	S	R	R	S	S	S	R	S
PG Nectariferous/10 g honey (rounding to tens of thousands)		140,000	170,000	80,000	70,000	30,000	100,000	170,000	110,000	110,000	60,000
HED/P		practically none	practically none	practically none	practically none	few	few	few	few	practically none	few
		practically none = 0,00–0,09						few = 0,10–0,9			

### 3.3 Physicochemical Analysis

The results of physicochemical analysis are shown in Table 3. All parameters were compliant with the requirements of European and International legislation (Council-Directive, 2001; CXS-12-1981, 1981), provided that heather honey “Αnama” is considered “honey with a low natural enzyme content”. Based on international regulations the diastase activity of honey is in general not less than 8 Schade units, and in the case of honeys with a low natural enzyme content not less than 3 Schade Units (CXS-12-1981, 1981). In the current study, Anama honey had a Diastase number of m.v. = 7.3 Schade units (SD = 3.2, range = 2.0–11.5 Schade units). All samples were freshly collected and the HMF value, checked for some of them, was always <15 mg/kg supporting the above categorization of Anama honey (Council-Directive, 2001). The low diastase content is not surprising, as it is also mentioned in the literature for Erica sp. honeys (Oddo et al., 2004; Thrasyvoulou et al., 2018).

**TABLE 3:** Physicochemical parameters of Anama honeys.

Sample	Colour^a^ (mm Pfund)	pH	Moisture (%)	Electrical conductivity (mS/cm)	Diastase (DN)	Fructose (g/100 g)	Glucose (g/100 g)	Saccharose (g/100 g)	Maltose (g/100 g)	Fructose + Glucose (g/100 g)	Fructose/Glucose ratio
IKF18_23	105	4.2	17.9	0.55	8.7	40.2	35.1	0.5	1.7	75.3	1.1
IKF18_24	105	4.2	18.3	0.54	8.3	39.6	34.5	0.5	1.9	74.1	1.1
IKΑ18_29	105	4.4	17.8	0.74	2.0	35.5	31.0	0.4	1.4	66.5	1.1
IKF19_22	100	4.0	18.4	0.38	9.5	37.5	31.6	0.3	2.6	69.1	1.2
IKF19_23	105	4.2	17.6	0.59	8.6	37.9	29.0	0.3	2.9	66.9	1.3
IKΑ19_27	105	4.5	17.1	0.95	3.2	34.1	27.4	0.0	0.9	61.4	1.2
IKΑ19_28	105	4.5	17.5	0.61	5.3	34.7	28.4	0.1	1.0	63.1	1.2
IKΑ19_30	105	4.4	18.7	0.97	5.7	34.7	29.3	0.0	1.0	64.0	1.2
IKF20_13	105	4.2	19.3	0.52	11.5	40.2	34.4	1.3	2.3	74.6	1.2
IKΑ20_14	105	4.5	17.4	0.83	10.6	35.8	29.6	0.6	1.9	65.4	1.2
mean	105	4.3	18.0	0.67	7.3	37.0	31.0	0.4	1.8	68	1.2
SD	1,6	0.2	0.7	0.20	3.2	2.4	2.8	0.4	0.7	5	0.1

a As long as it remains liquid. A fine crystallization occurs, usually 1–2 months after harvesting.

As regards the electrical conductivity of heather honey, it is considered an exception by International and Greek legislation and therefore there is no legal limit (Council-Directive, 2001; Oddo et al., 2004; Thrasyvoulou et al., 2018; Author Anonymous, 2022; CXS-12-1981, 1981; Thrasyvoulou and Manikis, 1995). Nevertheless, the electrical conductivity determined for “Anama” honey in the current study, is consistent with the values given by the International literature (e.g. m.v. = 0.67, SD = 0.16 mS/cm, 20°C (Council-Directive, 2001; Oddo et al., 2004; Thrasyvoulou et al., 2018; CXS-12-1981, 1981; Thrasyvoulou and Manikis, 1995), Table 3: m.v. = 0.67, SD = 0.20 mS/cm, 20°C).

Among the rest of physicochemical parameters, the relatively high percentage of moisture is worthy of note: m.v. = 18.0%w/w, SD = 0,7 (Table 3), being in agreement with International Legislation, as the upper legal limit of heather honey is, by way of exception 23%, instead of 20% for the rest of honeys [48. 49]. Sugar analysis was also conducted. The values determined for Fructose, Glucose, Saccharose, and Maltose were within the legal limits for blossom honey (Council-Directive, 2001; CXS-12-1981, 1981). The color of Anama honey is initially reddish-bronze with dark green tones (m.v. = 105 mm Pfund), but it soon turns into light caramel color, after a fine crystallization occurs, 1–3 months after harvesting. Finally, the determined pH values ranged from 4.0 to 4.5 (Table 3).

## 4 Discussion

### 4.1 Anama Honey Secondary Metabolites—Chemical Markers

The UHPLC-HRMS profile of the phenolic compounds of the three different monofloral honeys exhibited some common compounds including p-coumaric acid, caffeic acid, syringic acid, and flavones such as apigenin and chrysin and the flavanones, hesperetin, pinocembrin, taxifolin and naringenin (Table 4). These phenolic compounds are also common in various types of honeys as reported in the literature. More specifically, syringic acid and chrysin were previously reported as thyme, pine, and fir Greek honey components (Karabagias et al., 2014), displaying substantial fluctuation in concentrations among these types of honeys. In the presented study, though quantitation was not performed, considering the relative abundances obtained, such a marked concentration increase for one particular honey was not observed. Chrysin and pinocembrin were also key components of Portuguese heather honey (Ferreres et al., 1994). Apigenin, p-coumaric acid, and taxifolin were additionally reported as Greek heather and thyme honey constituents, demonstrating differences in concentrations (Koulis et al., 2021). Caffeic acid and hesperetin identified in the presented work, are also constituents of several honey types, including heather honey (Sergiel et al., 2014). Generally, heather honeys from Erica are characterized by high amounts of abscisic acid (Ferreres et al., 1996; da Silva et al., 2021) a fact also confirmed in this study. Quantitation of abscisic acid due to its indisputable utility as a chemical marker and the availability of analytical standard was performed in all Anama honey samples by LC-MS, resulting in a mean concentration of 104.11 ± 7.15 μg/g honey, (see Supplementary Figure S7 and Supplementary Materials S7, S8). Overall, this compound was reported to be used for floral authentication, but the quantitative analysis is crucial since abscisic acid is a constituent of another type of honeys at lower concentrations reaching the ng/g scale (Wang et al., 1998).

**TABLE 4:** Phenolic compounds common in Anama, Thyme and Pine honeys.

Compound annotation	Class	Monoisotopic Mass (Da) experimental	tR (min)	MS/MS fragment ions (*m/z*)	Molecular formula	Adduct ion
Apigenin	Flavones	270.0530	13.389	271.06/153.01	C_15_H_10_O_5_	[M+H]^+^
Caffeic acid	Hydroxycinnamic acid	180.0422	13.89	163.03	C_9_H_8_O_4_	[M+H]^+^
Chrysin	Flavones	254.0579	15.423	253.05/143.05	C_15_H_10_O_4_	[M-H]^−^
Hesperetin	Flavanones	302.0791	10.873	285.04/215.03	C_16_H_14_O_6_	[M-H]^−^
Naringenin	Flavanones	272.0686	12.422	271.06/151.00	C_15_H_12_O_5_	[M-H]^−^
p-Coumaric acid	Hydroxycinnamic acid	164.0464	8.295	119.05	C_9_H_8_O_3_	[M-H]^−^
Pinocembrin	Flavanone	256.0735	14.15	153.01/131.04	C_15_H_12_O_4_	[M+H]^+^
Syringic acid	Phenolic acid	198.0522	8.438	182.02/123.01/166.99	C_9_H_10_O_5_	[M-H]^−^
Taxifolin	Flavanone	304.0585	12.11	227.03/149.02	C_15_H_12_O_7_	[M-H]

The results of untargeted HRMS metabolomic analysis and subsequent workflow unveiled 32 compounds as potential chemical markers of Anama honey, most of which have never been reported as Greek heather honey components (Table 1). One of the differentially increased molecules in “Anama” honey was annotated as the compound picein (Table 1). Picein is a phenolic compound, glucoside of piceol, reported as a constituent of several plants. Among them, *Salvia* (Lamiaceae) (Wang et al., 1998), Opuntia ficus-indica (Cactaceae) (Strid, 2016), and Helianthemum (Cistaceae) (Chemam et al., 2017), are plants of high beekeeping value, widespread in Ikaria and Fournoi islands (Table 2). For the mass feature with *m/z* 332.1101, tR = 8.69 min, the molecular formula of C_14_H_20_O_9_ was assigned to leonuriside A (Table 1). Leonuriside A is a phenolic glycoside reported to be encountered in plants of Paronychia genus (native to Ikaria Island), one of the genera of the family Caryophyllaceae, while pollen grains of these nectarless plants have been identified in Anama honey samples in this work (Table 2). Astragalin, a kaempferol O-glucoside, detected at tR = 10.5 min with *m/z* 448.1007 (Table 1) has been also reported as a Polish heather honey constituent (Goslinski et al., 2021), while it is its first report in Greek heather honey. Kaempferol-3-Ο-galactoside (monoisotopic mass at m/z 448.1007, molecular formula C_21_H_20_O_11_, Table 1) or trifolin, is a kaempferol derivative identified in Anama honey. A respective glucoside was detected in carob fruit (*Ceratonia siliqua* L.) (Kyriacou et al., 2021). Pollen of Ceratonia is identified in nine out of ten samples of Anama honey, with a relatively high pollen frequency (Table 2). Salicylic acid, a phenolic acid detected at tR10.80 with m/z 138.0306), has been also reported in a previous study using gas chromatography–mass spectrometry, as a component of Greek thyme honey at relatively low concentrations (Alissandrakis et al., 2009). 2-anisic acid (2-methoxy-benzoic acid) detected at tR = 10.81 with m/z 152.0474 (Table 1), has been previously reported as a volatile marker for Greek cotton honey (Alissandrakis et al., 2005).

In the frame of rarely reported compounds, domesticoside (tR = 8.88, with m/z 344.1109, Table 1), a phenolic glycoside, was putatively annotated for the first time in Anama honey. This molecule is a component of several plants and among them *Artemisia* and *Prunus*. To our knowledge, two species of the genus *Artemisia* are distributed in Ikaria: *Artemisia arborescens* and *Artemisia vulgaris*. With regard to *Prunus*, there are several *Prunus* species, mostly cultivated, and abundant in Ikaria Island. Catalpol (tR = 8.63, with m/z 362.1205, Table 1) is an iridoid glycoside, of the class of monoterpenoids, described as a nectar secondary compound (Michaud et al., 2019). Another iridoid glycoside aucubin (tR = 11.04, with m/z 346.1258, Table 1), along with catalpol, are pharmacologically active natural components of numerous plants (Sertic et al., 2015; Miehe-Steier et al., 2015). Ganolucidic acid B (tR = 17.73, with m/z 502.3296, Table 1), belongs also to the terpenoids family and it has been isolated from Ganoderma Lucidum fungus. Its distinctive increase in Anama honey is worth mentioning considering its first report in honey, and the pronounced bioactivity of Ganoderma Lucidum which is conceived as traditional medicine (Yang Y. et al., 2019). Methyl 3-aminobenzoate (M3AB) (tR = 6.67, with m/z 151.0634, Table 1) belongs to aminobenzoates, while its isomer methyl 2-aminobenzoate or methyl anthralinate is established as a chemical marker of citrus honey (Sesta et al., 2008). Consequently, M3AB can be reported as a potential chemical marker of Anama honey. Benzoate derivatives are also key synthons for the development of bioactive molecules (Kronenberger et al., 2020).

Regarding alkyl phenols, 4-propylphenol (tR = 11.02, with m/z 136.0889, Table 1) emerged as a putative chemical marker of Anama honey. The literature search showed that the methyl ether of 2-propylphenol has been a component of honeydew honey (Štefániková et al., 2021). 3-Propylphenol and 4-propylphenol have been identified as aroma compounds of fermented cocoa (Füllemann and Steinhaus, 2020), the latter being an inhibitor of cyclic AMP phosphodiesterase (Nikaido et al., 1981). Another alkyl phenol, 2-ethylphenol (tR = 7.98, with m/z 122.0732, Supplementary Table S1), was also increased in Anama samples. Ethylphenols are reported as aroma compounds in wine (Monje et al., 2002).

Amino acids, including tryptophan, are also described as components of honey (Leu et al., 2012). D-tryptophan (tR = 10.81, with m/z 204.0899, Table 1) was detected in the Anama honey samples, whereas it has already been proposed as a discriminating marker of Acacia honey (Pirini et al., 1992). Tryptophan is pivotal in protein-protein interactions and its metabolite, kynurenic acid, displays significant bioactivity, being assigned as a putative biomarker of chestnut honey (Turski et al., 2016).

Benzaldehyde and its derivatives are well acquainted with volatile components of honey (Karabagias et al., 2019). 4-ethylbenzaldehyde (tR = 7.73, with m/z 134.0731, Table 1) can be utilized as a discriminating chemical compound of Anama. Benzaldehyde has been identified as a Greek heather honey constituent using GC-MS (Xagoraris et al., 2021), while its ethylated derivative has been detected for the first time in this work in Anama heather honey samples by LC-HRMS. Cuminaldehyde (tR = 16.43, with m/z 148.0889, Table 1), another benzaldehyde derivative, was differentially increased in Anama honey. This aldehyde is reported as a constituent of Croatian lime tree honey (Lusic et al., 2007) and Jarrah honey, another important antimicrobial commercial honey, originating from remote parts of Western Australia (Anand et al., 2019). Ferulic acid (tR = 8.90, with m/z 194.0580, Table 1) and its derivative, 5-hydroxyferulic acid methyl ester (tR = 11.21, with m/z 210.0524, Table 1) were also differentially increased in Anama honey (Table 1).

6-Methoxyluteolin 7-rhamnoside (tR = 11.54, with m/z 462.1163, Table 1) is a flavonoid reported in Brazilian red propolis (Silva-Beltran et al., 2021; do Nascimento et al., 2019), known especially for its biological potential (dos Santos et al., 2021). Isophorone (tR = 7.74, with m/z 138.1045, Table 1), a norisoprenoid compound, was identified in Anama honey samples while its MS spectrum and MS/MS fragments are presented in Figure 6. Norisoprenoid compounds, usually identified by means of gas chromatography, have been reported as constituents-markers of heather honey from Greece (Xagoraris et al., 2021) and other countries (Castro-Vázquez et al., 2012); hence, this work comes to verify previous findings. Isophorone has also been reported as a constituent of essential oils of *Eruca vesicaria*, a plant found in Aegean Islands (Hichri et al., 2019) and in *Crocus* species from Greece (Lamari et al., 2018). *Crocus* is widespread in Ikaria island and blooms simultaneously with *Erica manipuliflora*, during autumn and winter (Table 2).

The presented metabolomics approach also disclosed (+)-7-isomethyl jasmonate (isomethyl ester of jasmonic acid) as a characteristic compound that was found to increase in Anama honeys. Another key ester of jasmonic acid, methyl jasmonate (MJ), is a volatile compound known for its utility in plant defense mechanisms, and as an elicitor to improve the phenol content of fruits. It is found in higher plants, so its detection is expected. Jasmonates have long been studied for their anticancer activity. MJ, the most potent of this family, exhibits a specific cell death-induction effect on numerous cancer cells (Raviv et al., 2013). Vitamins are also reported as honey components. Riboflavin (tR = 9.27, with m/z 376.1384, Table 1) and its degradation product, lumichrome (tR = 10.81, with m/z 242.0802, Table 1) were differentially increased in Anama honey compared to thyme and pine honey of the same region. Both compounds have been reported as characteristic molecules of Dalmatian sage honey (Tuberoso et al., 2012). But, in principle, this finding is in agreement with the results regarding Turkish heather honey (Kıvrak, 2016), where vitamins of the B-group were predominant, and Greek heather honey (Koulis et al., 2021), in which lumichrome was abundant. An additional compound worth mentioning is dehypoxanthine futalosine (tR = 7.08, with m/z 296.0897, Table 1). It is a biosynthetic intermediate in the menaquinone (Vitamin K) biosynthesis (Manion-Sommerhalter et al., 2021), first reported as a potential honey constituent.

Phenylacetic acid (tR = 8.48, monoisotopic mass at m/z 166.063, Table 1) is a hydroxy monocarboxylic acid, hitherto stated as a potential marker of thistle honey (Tuberoso et al., 2011), found also in Manuka and Kanuka nectars (Bong et al., 2018), and typical for cornflower honey (Kus et al., 2014). The results of this work come to verify its role as a “marker substance” due to its accumulation in Anama honey (Koulis et al., 2021). 3,4-Dimethoxycinnamic acid (tR = 11.02, monoisotopic mass at m/z 208.0734, molecular formula C_11_H_12_O_4_), which was increased in Anama, is a molecule described as a marker of black locust (*Robinia pseudoacacia*) honey (Margaoan et al., 2021), also found in Greek cotton honey (Alissandrakis et al., 2005). The current work enlarges its utility as a non-specific chemical marker. Another constituent tentatively annotated in Anama honey is trichocarpin (tR = 12.4, monoisotopic mass at m/z 406.1277, Table 1). This molecule is referenced as a volatile metabolite of balsam poplar (a type of poplar tree) (Mattes et al., 1987), but is not yet reported as a honey component (based on research on the accessible bibliography). It is noteworthy that certain poplar trees are a pivotal source of propolis for bees. In the same context, plastoquinol annotated in Anama honey (Table 1) is the reduced form of plastoquinone (present in photosynthetic pathways) and a dominant antioxidant (Borisova-Mubarakshina et al., 2019).

The results of this study indicate that the excess of nectariferous (n = 44) and nectarless (n = 23) plants detected in the Anama honey can “guarantee” to a large extent the natural foundation of several of the phytochemicals detected. In all cases, blooming-flowering period information is essential when trying to connect the findings with the botanical origin and bees’ foraging. With all these in view, it can be inferred that there is still room for additional phytochemical analysis on a plethora of species that are either limitedly, scarcely, or not investigated at all.

The latter is a limitation of the presented work in the effort to associate the chemicals identified in honey with specific plants. This will be overcome by conducting a systematic study of the flora of Ikaria and simultaneous chemical analysis of the dominant plants of these islands. With these in view, heather honey from other regions of Greece can also be collected and analyzed along with Anama honey to unveil any potential differences as regards chemical markers and melissopalynological analysis.

Overall, Anama honey possesses a distinctive chemical profile that discriminates it from other important honeys of the Aegean region, an area with important biodiversity of flora and fauna (Archipelagos, 2022). This is supported by the fact that an increased relative abundance of more than 32 bioactive compounds was found in Anama honey compared to pine and thyme honey from the same geographical origin. From a chromatography point of view, it is evident that UPLC-HRMS has the capacity to detect compounds that are usually identified by gas chromatography, such as benzaldehydes and norisoprenoids. It is also apparent that the following procedure managed to extract a diversity of compounds covering a wide range of polarities. This is the first holistic report on the phytochemical profile of this exceptional Greek honey, embracing cutting-edge high-resolution mass spectrometry and statistical analysis reinforced by fundamental melissopalynological analysis. Based on reports from the literature, the majority of the annotated compounds that belong to various chemical classes have exhibited antimicrobial, antioxidant, and antiviral properties (Margaoan et al., 2021). Specifically, leonuriside A has been reported to inhibit the growth of human breast cancer MCF-7 cell line (Thai et al., 2017), also acting as a potential anti-diabetic agent (Nguyen et al., 2018). Trifolin is a bioactive molecule that induces apoptosis in human lung cancer cells (Kim et al., 2016). Catalpol’s tentative characterization and subsequent calculation of the differential increase in Anama honey deserves particular attention. Catalpol demonstrates therapeutic potential for central nervous system diseases, exemplified by Alzheimer and Parkinson (Zhang et al., 2021). Its activity is attributed to the inhibition of inflammation, antioxidant stress, improvement of mitochondrial dysfunction, and synaptic damage. An additional bioactive iridoid annotated in this work is aucubin. Its beneficial character is confirmed by various studies, exerting anti-nephrotoxic (Potacnjak et al., 2020; Potocnjak et al., 2021), anti-inflammatory (Olajide et al., 2020), and anti-osteoporotic effects (Li et al., 2020). Finally, ferulic acid, a common component in several types of honeys, and its analogues possess antioxidant properties and are tested in therapeutic schemes against several diseases (Paiva et al., 2013). Consequently, the newly reported molecules and the rest of the potential chemical markers can set the basis for further investigation of the biological activity of Anama honey.

### 4.2 Pine and Thyme Honey Secondary Metabolites

The results of the increased secondary metabolites and compounds found in Thyme and Pine honeys employing UHPLC-HRMS analyses in comparison with Anama honey (Anama vs. Thyme, Anama vs. Pine) are presented in Supplementary Table S2.

#### 4.2.1 Thyme

Citrusin C (Supplementary Table S2) is a phenolic glycoside putatively annotated for the first time in thyme honey. It is found in several plants, such as *Ducrosia* (aerial parts) (Morgan et al., 2014), and *Ononis* (aerial parts) (Mezrag et al., 2017). Other citrusins (A, B, D) are reported constituents of several plants such as *Artemisia* (Xiao et al., 2021), *Cortex* (Yang S. et al., 2019), *Buxus* (Saleem et al., 2019), and sweet oranges (Díaz et al., 2014). Citrusins exhibit anti-inflammatory (Yang S. et al., 2019) and antiviral activity (Tan et al., 2012).

Another bioactive molecule putatively annotated in thyme honey is syringin (Supplementary Table S2). It is a phenolic glycoside found in a multitude of foods and plants such as oats (Pretorius et al., 2021) and *Daphne* (Frezza et al., 2021; Ghanadian et al., 2020). It is a bioactive molecule displaying cardioprotective action in synergies with other compounds (Yao et al., 2021). (±)-Ribaline is a rare quinoline alkaloid (Supplementary Table S2), referenced as a constituent of *Zanthoxylum mayu* (Rutaceae family) (Barrera-Thomas, 2015). Gardenoside is a bioactive iridoid originating from Gardenia fruits. Norcoclaurine (Supplementary Table S2) is a 1-benzylisoquinoline that has displayed anti-HIV activity (Kashiwada et al., 2005), therefore its possible annotation increases the bioactive profile of any natural product; in this case, thyme honey. Its (S)-enantiomer displays partial neuroprotective activity (Peana et al., 2019).

#### 4.2.2 Pine

The LC-MS analysis of pine honey sample extracts also displayed several bioactive molecules, some of them first reported in this type of honey. Piceid (Supplementary Table S2), amongst others, is acknowledged as the major resveratrol derivative in grape juices (Romero-Pérez et al., 1999). Piceid and resveratrol are recognized for their anti-aging properties (Shen et al., 2017), hence their annotation in natural products is an added value. 10-epi-Eupatoroxin (Supplementary Table S2) is a sesquiterpene lactone whose cytotoxic activity is promising, as demonstrated by a quantitative structure-activity relationship (QSAR) study (Scotti et al., 2007). 3-methylsuberic acid is its first putative annotated report in pine honey. Suberic acid and derivatives are found in plants such as *Isatis tinctoria* L. (Hartleb and Seifert, 1995), *Viola odorata* (Jasim et al., 2018)*,* and *Dalbergia odorifera* (Sun et al., 2021). Interestingly, in pine honey, the biologically important 10-hyrodxydecenoic acid (royal jelly acid) was also differentially increased. A summary of the current findings for thyme and pine honey is being prepared to present the full results and possible correlation with plant sources.

### 4.3 Melissopalynological Analysis of Anama Honey

Despite the predominance of Erica pollen in all samples (av. 85%, range 63–99%, Supplementary Table S4), the study of the whole pollen spectra generates important information about the natural habitat of Ikaria and Fournoi and reflects the characteristics of the biome, which possesses a rich flora. As expected, the main contribution to nectar, apart from *Erica manipuliflora*, comes from plants that flower in the same season as heather, i.e., autumn and winter, especially madrones (Arbutus), carob trees (Ceratonia), asparagus, false yellowhead (Dittrichia), common ivy (Hedera), etc. (Table 2). In contrast, nectarless plants are dominated by those that flower in spring, indicating that they come from pollen stored in honeycombs. Thus, the families of Anacardiaceae, Cistaceae, Oleaceae, Scrophulariaceae, Fagaceae, and Fabaceae are dominant, although these families include spring and summer flowering plants. The only exception is the autumn-blooming Cyclamen sp. pollen, which is present in the 70% of the honey samples.

Nectariferous plants from previous blooming periods were also detected. A possible explanation is that they come from frames bearing honey and beebread from a spring or summer foraging, that has not been extracted (e.g., due to the presence of brood). This explains the high rates of plants from the Lamiaceae family that are observed in some cases, since these islands also produce excellent quality thyme honey. Pollen of *Myrtus* and *Styrax* is also distinguished, due to the wide distribution of these plants on the island. (Table 2, Supplementary Table S4). Pollen grains per 10 g of Anama honey, ranged from 50,000 to 210,000 (m.v. 103,314, SD = 45,310) and taking into account only nectariferous plants PG Nect./10 g ranged from 30,000 to 170,000 (m.v. 127,387, SD = 46,460) (Table 2, Supplementary Table S4). In addition to pollen grains, HDE were observed and counted. According to Louveaux et al. (Louveaux et al., 1978), the extent to which a given honey sample is derived from different plant sources can be deduced from the frequencies of the pollens and honeydew elements in it. This rule is valid only if the honey contains few honeydew elements (HDE/*p* < 1). In our study, the ratio of honeydew elements to pollen grains was determined and found to be HDE/*p* < 1 in all cases (Table 2, Supplementary Table S4).

Therefore, apart from proving the main botanical origin of honey, the melissosopalynological analysis of honey “Anama” can provide information about its geographical origin, as it is directly related to the flora of Ikaria and Fournoi. Both nectariferous and nectarless plants contribute to the Melissopalynological profile of the honey studied.

### 4.4 Melissopalynological Analysis of Thyme and Pine Honey

#### 4.4.1 Thyme Honey

Thyme honey originating from Kea, Syros, and Fournoi islands, is dominated by pollen of Thymbra capitata (m.v. = 65%, range = 18%–97%), accompanied by pollen of the native flora of the Mediterranean maquis.

The most important families of nectariferous plants, observed in more than half of the samples, are reported here, along with some representative plant genera (in descending order of occurrence in honey): Lamiaceae (*Thymbra/Thymus, Salvia, Lavandula, Teucrium, Ballota, Phlomis/Lamium, Satureja*), Asteraceae (*Taraxacum/Crepis, Centaurea spinosa, Dittrichia/Inula, Anthemis*), Fabaceae (*Melilotus, Anthyllis hermanniae, Trifolium, Ononis, Acacia, Lotus, Vicia*), Brassicaceae (*Sinapis, Eruca*), Apiaceae (*Daucus/Crithmum//Foeniculum, Smyrnium, Ferula, Tordyllium, Pimpinella*), Boraginaceae (*Echium, Cynoglossum, Heliotropium*), Myrtaceae (*Eucalyptus, Myrtus*), Convolvulaceae (*Convolvulus*), Asphodelaceae (*Asphodelus*), Malvaceae (*Malva*), Aizoaceae, Oxalidaceae (*Oxalis*), Tamaricaceae [*Tamarix* (SYROS)], Rosaceae (KEA), Ericaceae [*Erica* (in specific cases as secondary pollen in FOURNOI thyme honey)], Aceraceae (*Acer,* in specific cases as secondary pollen, only in KEA thyme honey)].

As a result of quantitative analysis, the pollen grains per 10 g of thyme honey had a mean value of 29,000 (range = 3800–67,000). The number of honeydew elements was in all cases low or very low: HDE/*p* < 0.1 in 96% of samples.

#### 4.4.2 Pine Honey

Although pine honey comes from the honeydew of *Marchalina hellenica*, an insect that lives in the Pinus brutia forests of Aegean islands, melissopalynological analysis revealed a plethora of plants represented by the pollen encountered in the honey sediment. The mean value of taxa per sample was 41, range = 26–55, reflecting the rich flora of Samos island. Pine honey originating from Samos island is dominated by pollen of forest plants, especially deciduous trees, shrubs, climbing plants, and wildflowers. The most important families and plant genera, observed in more than half of the samples, are reported here, in descending order of occurrence in honey.

Families of nectariferous plants: Asteraceae, Fabaceae, Lamiaceae, Rosaceae, Ericaceae, Fagaceae, Boraginaceae, Asparagaceae, Apiaceae, Caesalpiniaceae, Brassicaceae, Araliaceae, Anacardiaceae, Myrtaceae, Smilacaceae, Lauraceae, Liliaceae, Styracaceae, Oxalidaceae.

Important Genera of nectariferous plants: *Castanea, Dittrichia,Inula, Rhus, Myrtus, Melilotus, Thymbra,Thymus, Erica, Asparagus, Hedera, Ceratonia, Taraxacum, Rubus, Anthemis, Sinapis, Trifolium, Smilax, Styrax, Laurus, Onopordum, Oxalis*.

Nectarless plants also play an important role in the melissopalynological profile of honeydew honey, especially considering that the honeydew itself does not provide pollen to bees. Therefore, families and genera of nectarless plants are listed below in descending order: Fagaceae (*Quercus coccifera/ilex, Quercus pubescens*), Cistaceae, Anacardiaceae (*Pistacia*), Oleaceae (*Olea*), Scrophulariaceae (*Verbascum*), Pinaceae, Hypericaceae (*Hypericum*), Fabaceae (*Calicotome-Genista*), Chenopodiaceae/Amaranthaceae, Ephedraceae (*Ephedra*), Platanaceae (*Platanus*), Rosaceae (*Poterium*), Ranunculaceae (*Anemone*).

As expected, the number of honeydew elements was numerous (HDE/*p* = 3.0–4.5) or very numerous (HDE/*p* > 4.5) in the 67% of the samples, and of medium quantity (HDE/*p* = 1.5–2.9) in the 30% of the samples. As a result of quantitative analysis, the pollen grains per 10 g of pine honey had a mean value of 71,000 (range = 13,000–240,000) and the HDE per 10 g of honey had a mean value of 245,000 (range = 100,000–570,000).

It is obvious that the pollen spectrum in heather, thyme, and pine honey differs significantly due to the different botanical origins and the different periods of the year in which the honey is harvested. Nevertheless, it is the comparative metabolomic analysis that revealed important metabolites and bioactive compounds for each type of honey.

## Conclusion

The application of an untargeted UHPLC-HRMS screening with a metabolomics workflow assisted by statistical analysis, provided a diversity of compounds as tentative, specific, and non-specific, chemical markers of Ikaria’s unique heather honey called “Anama”. 32 chemicals (among them, phenolic compounds and triterpenes) emerged as potential chemical markers of Anama honey, discriminating it from the other important Greek honeys of pine and thyme. Some of these chemicals, such as aucubin, catalpol, leonuriside A, picein, domesticoside, 4-ethylbenzaldehyde, 3,4-dimethoxycinnamic acid, dehypoxanthine futalosine, ganolucidic acid B, kaempferol-3-O-galactoside, 2-ethylphenol, and 4-propylphenol were not previously reported in Greek heather honey, and these compounds could be useful markers of the botanical origin of Anama which has the potential to be regarded as honey with protected designation of origin. Melissopalynological analysis also identified an excess of nectariferous (n = 44) and nectarless (n = 23) plants in Anama honey, and an attempt was made, in addition to the accurate HRMS spectra, to link the metabolites identified with the findings of the melissopalynological analysis. The latter is essential in the context of connecting secondary metabolites and compounds with the flora of this Mediterranean blue zone region, reinforcing the dependence of these compounds on the botanical origin of Anama honey. The identified metabolites exhibit significant antioxidant activity, offering Anama honey exceptional characteristics with advantageous effects on human health. The future perspective and steps of this work should include the targeted UHPLC-HRMS quantitative analysis of all commercially available chemical markers tentatively identified in this work. In the same context, nectar and pollen from the dominant plants of the region, which were identified in the presented study by extensive melissopalynological analysis, should be collected and subsequent phytochemical analysis pursued to fully elucidate the origin of the chemical substances found in Anama honey. Finally, heather honey from other regions of Greece can also be investigated in parallel with Anama honey to disclose any probable differences with respect to chemical markers and melissopalynological analysis.

## Data Availability

The original contributions presented in the study are included in the article/Supplementary Material. Further inquiries can be directed to the corresponding authors.
