# Supplementary material for: Characterization of Ikaria Heather Honey by Untargeted Ultrahigh-Performance Liquid Chromatography-High Resolution Mass Spectrometry Metabolomics and Melissopalynological Analysis
Source: Front Chem. 2022 Jul 22;10:924881. doi: 10.3389/fchem.2022.924881 (PMC9353074; doi:10.3389/fchem.2022.924881)
Supplement: Supplementary file 1 [file DataSheet1.docx]

Supplementary Material

**Characterization of Ikaria Heather Honey by Untargeted Ultrahigh-Performance Liquid Chromatography-High Resolution Mass Spectrometry (UHPLC-HRMS) Metabolomics and Melissopalynological Analysis**

Konstantinos M. Kasiotis,^1,*^ Eirini Baira,^1^ Styliani Iosifidou,^2^ Kyriaki Bergele,^2^ Electra Manea-Karga,^1^ Ioannis Theologidis,^1^ Theodora Barmpouni,^1^ Despina Tsipi,^2,*^ and Kyriaki Machera^1^

^1^ Laboratory of Pesticides’ Toxicology, Benaki Phytopathological Institute, 8 St. Delta Street, Kifissia, 14561 Athens, Greece

^2^ General Chemical State Laboratory, Independent Public Revenue Authority (A.A.D.E) , 16 An. Tsocha Street, Athens 115 21, Greece

***Corresponding authors:**

**Dr. Konstantinos M. Kasiotis,** Research Director, Benaki Phytopathological Institute, Department of Pesticides Control and Phytopharmacy, Laboratory of Pesticides’ Toxicology, 8 St. Delta Street, Athens, Kifissia 14561, Greece. E-mail: [K.Kasiotis@bpi.gr](mailto:K.Kasiotis@bpi.gr), Tel: +30 210 8180357

**Dr. Despina Tsipi**, Director of A Division, General Chemical State Laboratory, Independent Public Revenue Authority (A.A.D.E) , 16 An. Tsocha Street, Athens 115 21, Greece. E-mail: [d.tsipi@aade.gr](mailto:d.tsipi@aade.gr)Tel: +30 210 8180201

**Contents**

1. **Loading plots of PCA and OPLS-DA, Supplementary Figures 1-6**
2. **Supplementary Table 1.** Final score from the matching of *in silico* fragments from candidate molecules from Pubchem and Kegg databases against mass to charge values of differentially increased compounds of Anama honey, using the open source software MetFrag.
3. **Supplementary Table 2.** Putative annotation of differentially increased secondary metabolites and compounds in Thyme and Pine honeys after respective comparisons with Anama honey (Anama *vs* Thyme, Anama *vs* Pine) employing UHPLC-HRMS analyses. For the putatively annotation of the compounds databases such as mzCloud and Metlin were used. Moreover, the open source software MetFrag was employed matching in silico fragments from candidate molecules from Pubchem and Kegg databases against mass to charge values, using 5 ppm for candidate selection and 0.001 mass deviation to match generated fragments against MS/MS peaks.
4. **Supplementary Table 3.** Final score from the matching of in silico fragments from candidate molecules from Pubchem and Kegg databases against mass to charge values of differentially increased compounds of Thyme and Pine honeys, using the open source software MetFrag
5. **Supplementary Table 4.** Pollen spectrum of honeys studied. Data presented as relative frequencies (%) of pollen
6. **Supplementary Figure 7**
7. **UHPLC-HRMS quantitative analysis of abscisic acid**
8. **HPLC-PDA-ESI/MS separation of abscisic acid isomers**

**1. Loading plots of PCA and OPLS-DA**


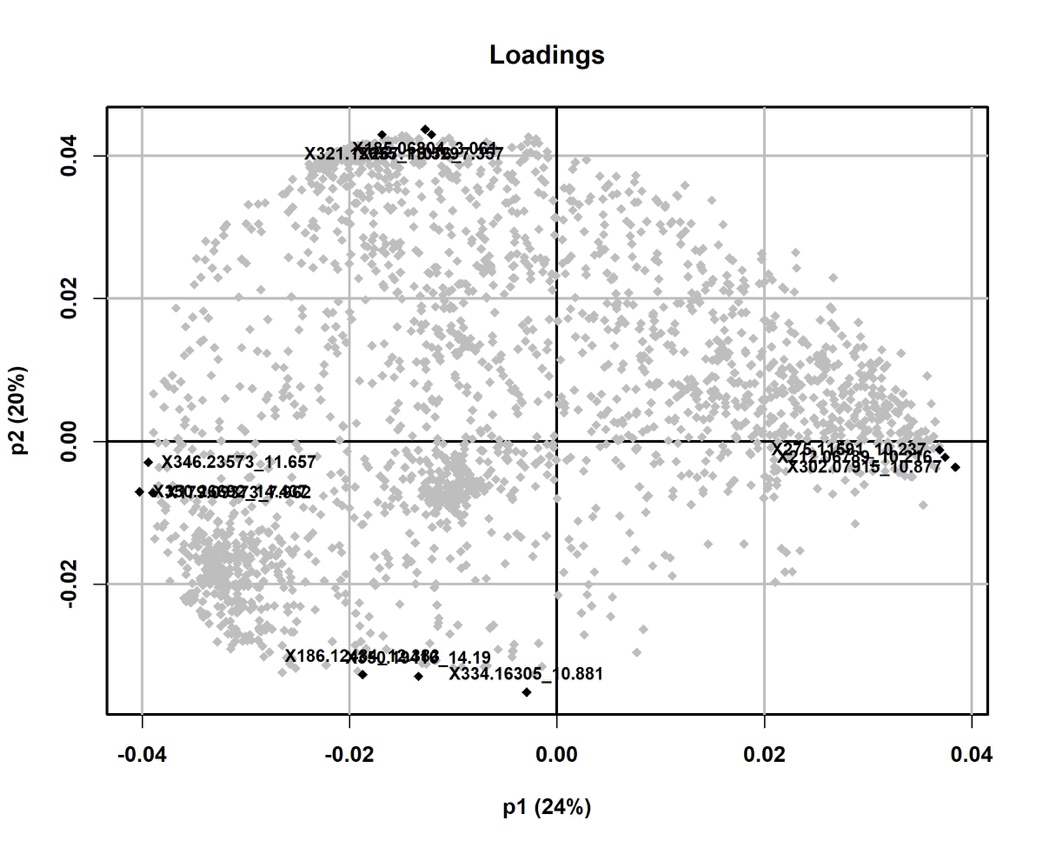


**Supplementary Figure 1. PCA negative loadings plot**


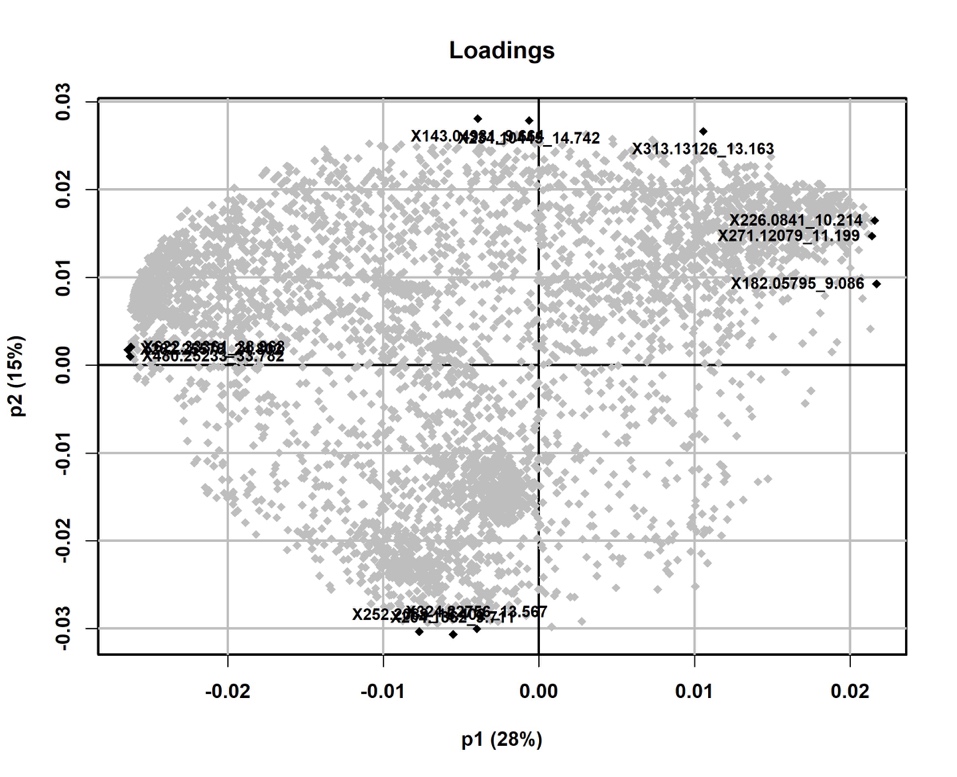


**Supplementary Figure 2. PCA positive loadings plot**


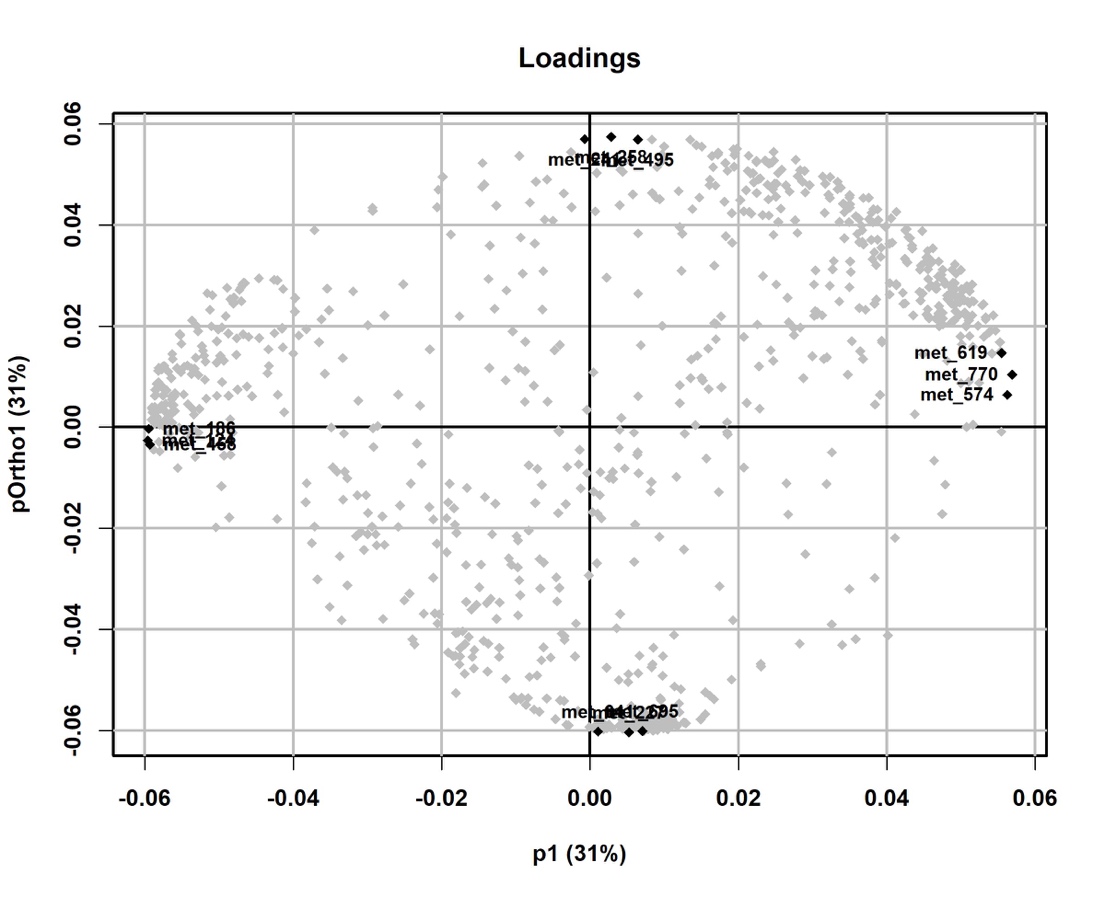


**Supplementary Figure 3. OPLS-DA pine negative loadings plot**


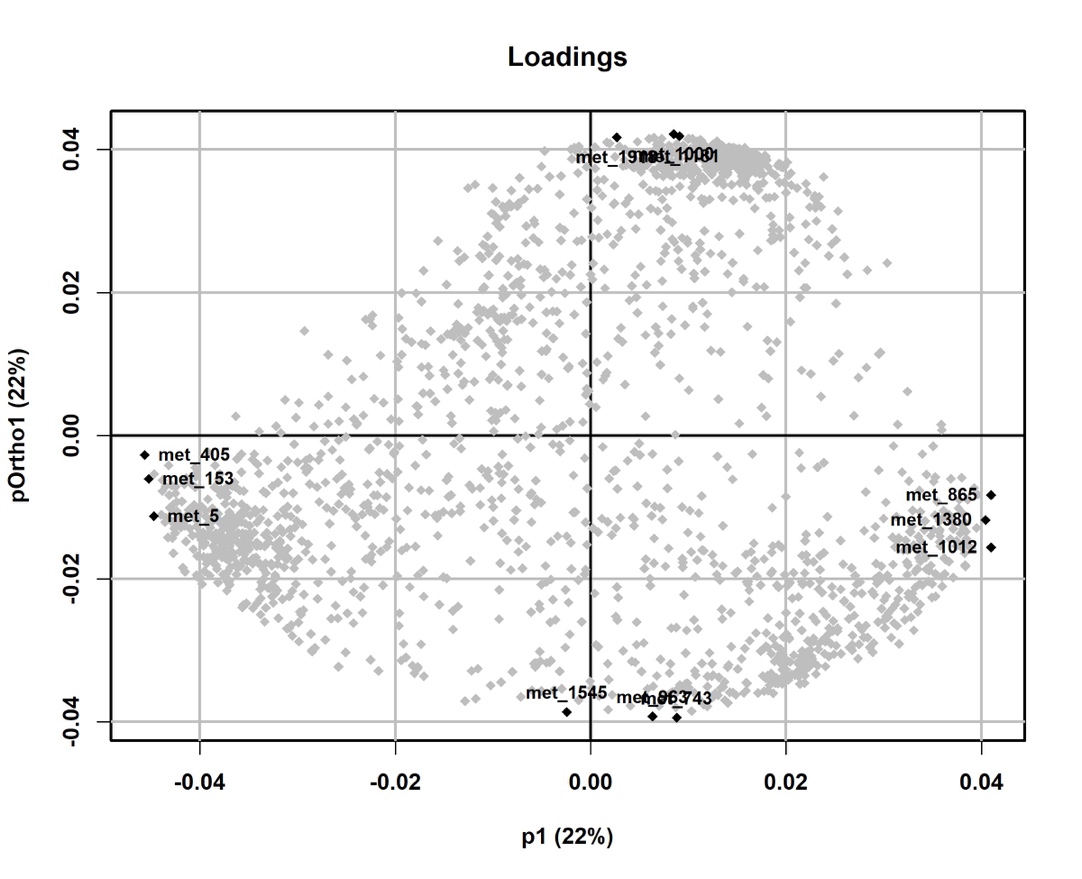


**Supplementary Figure 4. OPLS-DA pine positive loadings plot**


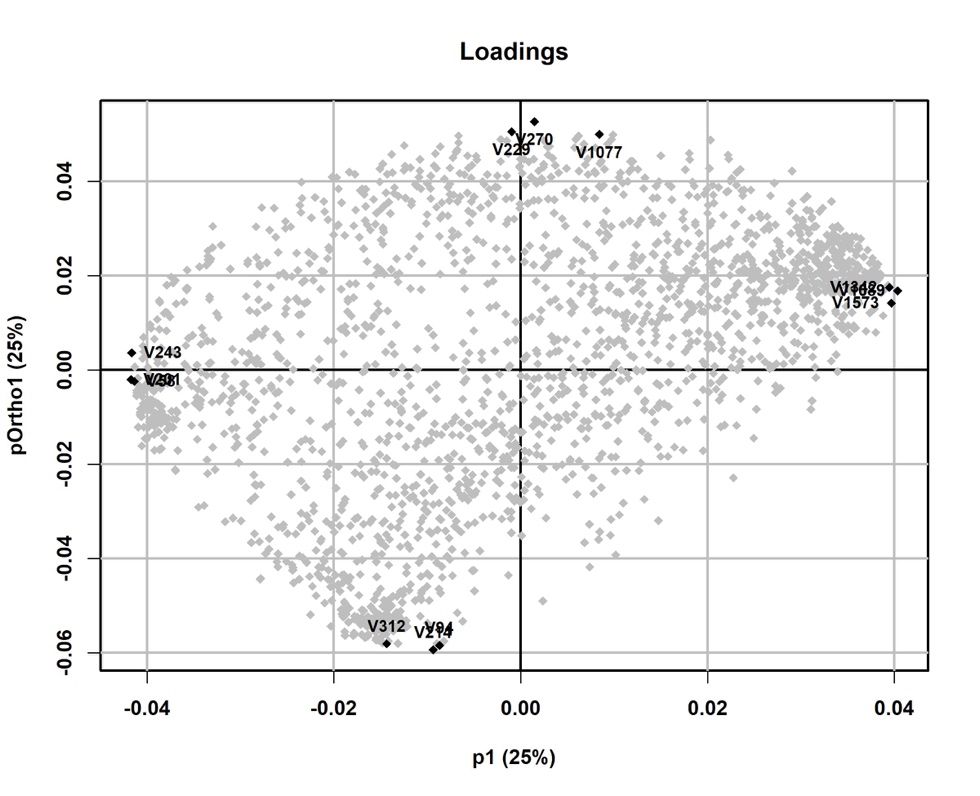


**Supplementary Figure 5. OPLS-DA thyme negative loadings plot**

**
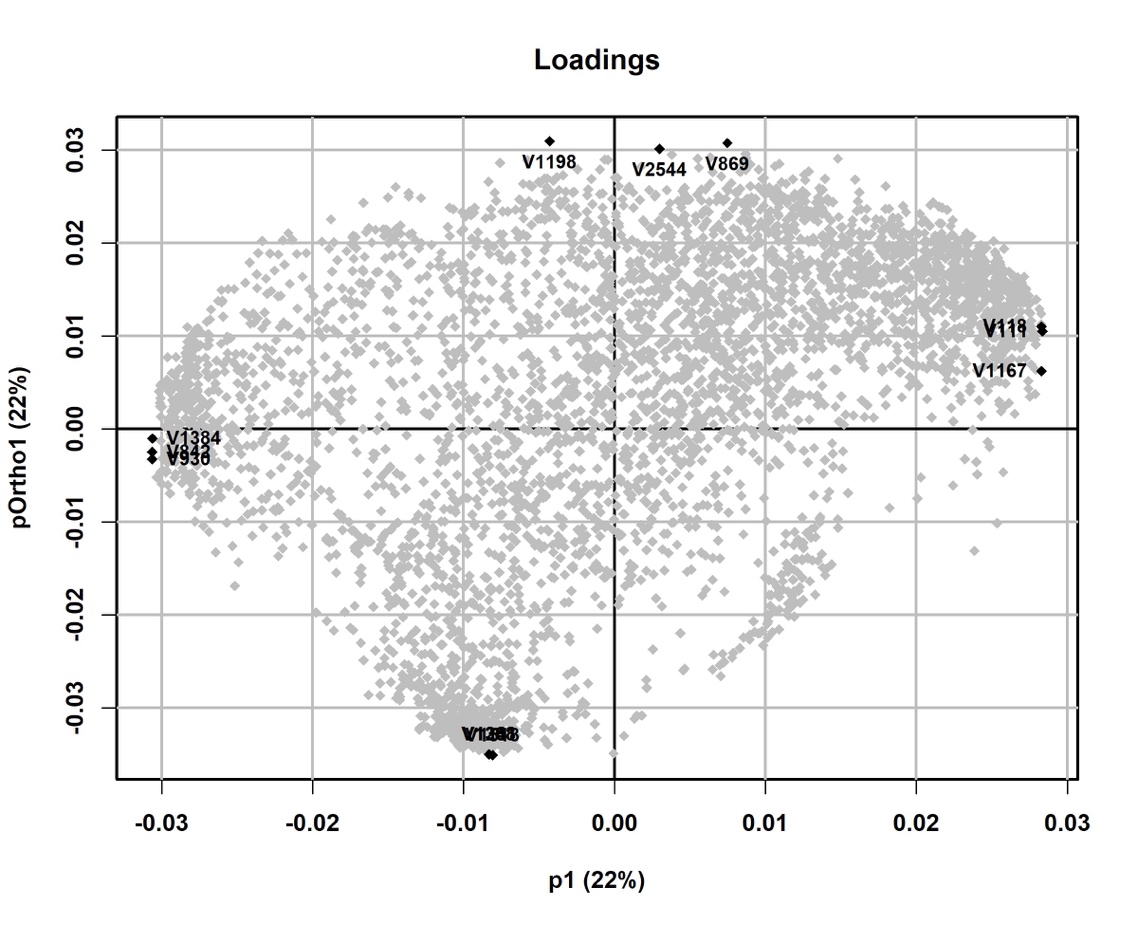
**

**Supplementary Figure 6. OPLS-DA thyme positive loadings plot**

**2. Supplementary Table 1.** Final score from the matching of *in silico* fragments from candidate molecules from Pubchem and Kegg databases against mass to charge values of differentially increased compounds of Anama honey, using the open source software MetFrag.

| **Compound Name** | **Final Score** |
| --- | --- |
| Aucubin | 0.878 |
| Catalpol | 0.925 |
| Dehypoxanthine futalosine | 1 |
| Domesticoside | 1 |
| 5-Hydroxyferulic acid methyl ester | 1 |
| (+)-7-Isomethyljasmonate | 1 |
| Kaempferol-3-O-galactoside | 0.848 |
| Leonuriside A | 1 |
| Ganolucidic acid B | 1 |
| 6-Methoxyluteolin 7-rhamnoside | 1 |
| p-Coumaroyl-D-glucose | 1 |
| 2-Ethylphenol | 1 |
| Phenyllactic acid | 0.960 |
| Picein | 1 |
| Plastoquinol-1 | 1 |
| 4-Propylphenol | 1 |
| Quercetin 3'-methyl ether 3-rhamnosyl-(1->2)-[glucosyl-(1->6)-glucoside] | 1 |
| Riboflavin | 1 |
| Trichocarpin | 1 |

**3. Supplementary Table 2.** Putative annotation of differentially increased secondary metabolites and compounds in Thyme and Pine honeys after respective comparisons with Anama honey (Anama *vs* Thyme, Anama *vs* Pine) employing UHPLC-HRMS analyses. For the putative annotation of the compounds databases such as mzCloud and Metlin were used. Moreover, the open source software MetFrag was employed matching *in silico* fragments from candidate molecules from Pubchem and Kegg databases against mass to charge values, using 5 ppm for candidate selection and 0.001 mass deviation to match generated fragments against MS/MS peaks.

| **Compound Annotation** | **Class** | **Monoisotopic Mass (Da) Experimental** | **tR (min)** | **MS/MS Fragment ions (*m/z*)** | **Molecular Formula** | **Adduct Ion** | **Differentially increased** |
| --- | --- | --- | --- | --- | --- | --- | --- |
| Citrusin C | Phenolic glycoside | 326.1368 | 8.40 | 71.01/101.02/116.04 | C_16_H_22_O_7_ | [M-H]^-^ | Thyme |
| Gardenoside | Iridoid | 404.1319 | 8.89 | 75.01/59.01 | C_17_H_24_O_11_ | [M-H]^-^ | Thyme |
| Syringin | Phenolic glycoside | 372.1421 | 9.55 | 71.01/59.01/89.02 | C_17_H_24_O_9_ | [M-H]^-^ | Thyme |
| Acrophylline | Alkaloid | 283.1208 | 13.56 | 120.08/166.08 | C_17_H_17_NO_3_ | [M+H]^+^ | Thyme |
| (±)-Ribaline | Alkaloid | 275.1159 | 10.23 | 84.04/230.11 | C_15_H_17_NO_4_ | [M-H]^-^ | Thyme |
| (R)-Norcoclaurine | Isoquinoline | 271.1207 | 7.38 | 108.04/80.04 | C_16_H_17_NO_3_ | [M+H]^+^ | Thyme |
| 2,3-Dihydroflavone | Flavonoid | 224.0838 | 13.48 | 103.05/121.02 | C_15_H_12_O_2_ | [M+H]^+^ | Thyme |
| Piceid | Stilbenoid glucoside | 390.1317 | 8.64 | 150.03/134.03/165.05 | C_20_H_22_O_8_ | [M-H]^-^ | Pine |
| 10-epi-Eupatoroxin | Sesquiterpenoid | 392.1474 | 9.35 | 150.03/195.06/165.05 | C_20_H_24_O_8_ | [M-H]^-^ | Pine |
| 3-Methylsuberic acid | Dicarboxylic acid derivative | 188.1048 | 11.33 | 125.09/97.10 | C_9_H_16_O_4_ | [M+H]^+^ | Pine |
| 10-HDA | Fatty acid | 186.1256 | 13.91 | 81.06/67.05 | C_10_H_18_O_3_ | [M+H]^+^ | Pine |
| Abietic acid | Terpenoid | 302.2245 | 28.09 | 121.10/107.08/93.06 | C_20_H_30_O_2_ | [M+H]^+^ | Pine |

**4. Supplementary Table 3.** Final score from the matching of *in silico* fragments from candidate molecules from Pubchem and Kegg databases against mass to charge values of differentially increased compounds of Thyme and Pine honeys, using the open source software MetFrag.

| **Compound Name** | **Final Score** | **Differentially increased** |
| --- | --- | --- |
| Citrusin C | 1 | Thyme |
| Gardenoside | 1 | Thyme |
| Syringin | 1 | Thyme |
| Acrophylline | 0.994 | Thyme |
| (±)-Ribaline | 1 | Thyme |
| (R)-Norcoclaurine | 0.886 | Thyme |
| Piceid | 0.977 | Pine |
| 10-epi-Eupatoroxin | 0.821 | Pine |
| 3-Methylsuberic acid | 1 | Pine |

**5. Supplementary Table 4.** Pollen spectrum of honeys studied. Data presented as relative frequencies (%) of pollen

For the calculation of the pollen frequency of nectariferous plants, nectarless plants are excluded

| **NECTARIFEROUS PLANTS (N)** | | **Samples** | | | | | | | | | |  |  |  |
| --- | --- | --- | --- | --- | --- | --- | --- | --- | --- | --- | --- | --- | --- | --- |
| **Family** | **Genus** | **IKF18-23** | **IKF18-24** | **IKA18-29** | **IKF19-22** | **IKF19-23** | **IKA19-27** | **IKA19-28** | **IKA19-30** | **IKF20-13** | **IKA20-14** | **No of samples** | **Average (%pollen)** | **s** |
| Aizoaceae | Carpobrotus |  |  |  |  |  |  | <1.0 | <1.0 |  |  | 2 |  |  |
| Apiaceae |  |  |  |  | <1.0 | 1.7 | <1.0 | <1.0 | <1.0 |  | <1.0 | 6 |  |  |
| Araliaceae | Hedera |  |  |  |  |  | <1.0 | <1.0 | <1.0 |  | 4.2 | 4 | 1.5 | 1.8 |
| **Asparagaceae** | **Asparagus** | <1.0 | <1.0 | 1.5 |  | 1.7 | 1.1 | 1.3 | 1.4 |  | 1.3 | **8** | 1.1 | 0.6 |
| Asteraceae | Anthemis |  |  |  |  | <1.0 |  |  | <1.0 |  |  | 2 |  |  |
| Asteraceae | Carthamus |  |  |  | <1.0 |  |  |  |  |  |  | 1 |  |  |
| Asteraceae | Centaurea raphanina T |  |  |  | <1.0 |  |  | <1.0 | <1.0 | <1.0 |  | 4 |  |  |
| Asteraceae | Centaurea solstitialis T |  |  |  |  |  |  |  | <1.0 |  |  | 1 |  |  |
| **Asteraceae** | **Dittrichia / Inula** | <1.0 | 1.0 |  | <1.0 | 3.0 |  | <1.0 | <1.0 | <1.0 |  | **7** | 0.7 | 1.0 |
| Asteraceae | Onopordum |  |  |  |  |  | <1.0 | <1.0 | <1.0 | <1.0 | <1.0 | 5 |  |  |
| Asteraceae | Taraxacum |  |  | <1.0 | <1.0 | <1.0 | <1.0 |  | <1.0 |  | <1.0 | 6 |  |  |
| Brassicaceae | Sinapis |  |  | <1.0 |  |  |  | <1.0 |  |  | <1.0 | 3 |  |  |
| Cactaceae | Opuntia |  |  |  | <1.0 |  |  |  |  |  |  | 1 |  |  |
| **Caesalpiniaceae** | **Ceratonia** | 11.7 | 11.0 | <1.0 |  | 5.1 | <1.0 | 1.3 | 1.6 | <1.0 | <1.0 | **9** | 3.5 | 4.7 |
| Campanulaceae |  |  |  |  | <1.0 |  |  |  | <1.0 |  |  | 2 |  |  |
| Colchicaceae | Colchicum |  |  |  |  |  |  |  |  |  | <1.0 | 1 |  |  |
| Convolvulaceae | Convolvulus |  |  |  | 2.0 | <1.0 |  |  |  | <1.0 | <1.0 | 4 | 0.7 | 0.9 |
| Cucurbitaceae | Cucurbita |  |  |  |  |  |  |  | <1.0 |  |  | 1 |  |  |
| **Ericaceae** | **Arbutus** | <1.0 |  | 1.1 |  |  | <1.0 | <1.0 | <1.0 | <1.0 | 1.0 | **7** | 0.5 | 0.4 |
| **Ericaceae** | **Erica** | 86 | 88 | 96 | 63 | 63 | 84 | 91 | 91 | 99 | 89 | **10** | **85** | **12.4** |
| Fabaceae | Acacia |  |  |  |  |  |  |  | <1.0 |  | <1.0 | 2 |  |  |
| Fabaceae | Anthyllis hermanniae T |  |  |  | <1.0 |  |  |  |  |  |  | 1 |  |  |
| Fabaceae | Trifolium pratense T |  |  | <1.0 |  |  |  | <1.0 |  |  |  | 2 |  |  |
| Fabaceae | Vicia |  |  |  |  |  |  |  |  |  | <1.0 | 1 |  |  |
| Fabaceae (Leguminosae) |  |  |  |  |  |  | <1.0 | <1.0 | <1.0 |  |  | 3 |  |  |
| Fagaceae | Castanea |  |  |  |  |  |  |  |  |  | 1.0 | 1 | 1.0 |  |
| Hyacinthaceae | Drimia |  |  |  |  |  |  |  |  |  | <1.0 | 1 |  |  |
| Iridaceae | Crocus |  |  | <1.0 |  |  | <1.0 |  | <1.0 |  | <1.0 | 4 |  |  |
| Lamiaceae | Ballota |  |  |  |  | 1.3 |  |  |  |  |  | 1 | 1.3 |  |
| **Lamiaceae** | **Lavandula** | <1.0 |  | <1.0 |  | <1.0 | <1.0 | 1.6 | 1.0 |  | <1.0 | **7** | 0.6 | 0.5 |
| Lamiaceae | Ocinum | <1.0 |  |  |  |  |  |  |  |  |  | 1 |  |  |
| Lamiaceae | Salvia |  |  |  |  |  |  |  |  | <1.0 |  | 1 |  |  |
| Lamiaceae | Satureja |  |  |  |  |  |  | <1.0 | <1.0 |  |  | 2 |  |  |
| Lamiaceae | Teucrium |  |  |  | <1.0 | <1.0 |  |  |  |  |  | 2 |  |  |
| **Lamiaceae** | **Thymbra / Thymus** | <1.0 | <1.0 |  | 33.6 | 22.0 |  | 1.5 | <1.0 | <1.0 | <1.0 | **8** | 7.4 | 13.0 |
| Lauraceae | Laurus / Persea |  |  |  |  |  |  |  | <1.0 |  |  | 1 |  |  |
| Liliaceae |  |  |  |  |  |  |  | <1.0 | <1.0 |  |  | 2 |  |  |
| Malvaceae |  |  |  |  |  |  |  |  |  |  | <1.0 | 1 |  |  |
| Myrtaceae | Myrtus |  |  |  |  |  | 8.7 | <1.0 | <1.0 |  |  | 3 | 3.2 | 4.7 |
| Oxalidaceae | Oxalis |  |  | <1.0 |  |  | <1.0 | <1.0 | <1.0 |  |  | 4 |  |  |
| Ranunculaceae | Clematis |  |  |  |  |  |  |  |  |  | <1.0 | 1 |  |  |
| Rosaceae | Pyrus-Prunus |  |  | <1.0 |  |  | <1.0 |  |  |  |  | 2 |  |  |
| Styracaceae | Styrax |  |  | <1.0 |  |  | 2.5 | <1.0 | <1.0 |  | <1.0 | 5 | 0.7 | 1.0 |
| Zygophyllaceae | Tribulus | <1.0 |  |  |  |  |  |  |  |  |  | 1 |  |  |
| **NECTARLESS PLANTS (NL)** | | **Samples** | | | | | | | | | |  |  |  |
| **Family** | **Genus** | **IKF18- 23** | **IKF18- 24** | **IKA18- 29** | **IKF19- 22** | **IKF19- 23** | **IKA19- 27** | **IKA19- 28** | **IKA19- 30** | **IKF20- 13** | **IKA20- 14** | **No of samples** | **Average (% pollen)** | **s** |
| **Anacardiaceae** | **Pistacia** | <1.0 | <1.0 | 1.9 | <1.0 | <1.0 | 2.6 | 1.0 | 1.5 | 1.7 | 6 | **10** | 1.6 | 1.8 |
| Arecaceae (Palmae) |  | <1.0 | <1.0 |  |  |  |  |  |  |  |  | 2 |  |  |
| Betulaceae |  |  |  |  |  |  |  |  | <1.0 |  | <1.0 | 2 |  |  |
| Caryophyllaceae |  |  |  |  |  |  | <1.0 |  | <1.0 |  |  | 2 |  |  |
| Chenopodiaceae/Amaranthaceae |  |  |  |  |  |  |  |  | <1.0 |  |  | 1 |  |  |
| **Cistaceae** |  |  | <1.0 | <1.0 | <1.0 | 1.9 | 17.7 | 7.3 | 9.3 | <1.0 | 2.1 | **9** | 4.4 | 6.0 |
| Cupressaceae |  | <1.0 | <1.0 | <1.0 |  | <1.0 |  |  |  | 1.3 |  | 5 |  |  |
| **Fabaceae** | **Calicotome-Genista** | <1.0 | <1.0 | <1.0 | 3.2 | 20 |  |  | <1.0 |  |  | **6** | 4.1 | 7.9 |
| **Fagaceae** | **Quercus coccifera Τ** |  | <1.0 | 6.6 |  | 1.3 | 13.9 | 7.9 | 5.6 | <1.0 | 16.2 | **8** | 6.5 | 6.1 |
| Fagaceae | Quercus ithaburensis Τ |  |  |  |  |  |  | <1.0 | <1.0 |  | 1.0 | 3 | 0.8 | 0.2 |
| Juglandaceae | Juglans |  |  | <1.0 |  |  |  |  | <1.0 |  | <1.0 | 3 |  |  |
| Oleaceae | Fraxinus |  |  |  |  |  | <1.0 |  |  |  |  | 1 |  |  |
| **Oleaceae** | **Olea** | <1.0 | <1.0 | <1.0 | 23.6 | 3.6 | 3.2 | 1.2 | 0.9 | <1.0 | 1.9 | **10** | 3.7 | 7.1 |
| Papaveraceae | Papaver |  |  |  | <1.0 |  |  |  |  |  |  | 1 | 0.9 |  |
| Pinaceae |  |  | <1.0 | <1.0 |  | <1.0 | <1.0 | <1.0 | <1.0 |  |  | 6 |  |  |
| Plantaginaceae | Plantago |  |  |  |  |  |  |  |  |  | <1.0 | 1 |  |  |
| Platanaceae | Platanus |  |  |  |  |  |  |  |  |  | <1.0 | 1 |  |  |
| Polygonaceae | Rumex |  | <1.0 |  | <1.0 |  |  |  | <1.0 |  |  | 3 |  |  |
| **Primulaceae** | **Cyclamen** | <1.0 | <1.0 | 1.3 |  | 1.0 |  |  |  | 2.1 | 1.0 | **6** | 1.0 | 0.7 |
| Rafflesiaceae | Cytinus |  |  | <1.0 |  |  |  |  |  |  |  | 1 |  |  |
| Ranunculaceae | Ranunculus |  |  |  |  | <1.0 |  | <1.0 |  |  | <1.0 | 3 |  |  |
| Rubiaceae | Galium |  |  |  |  |  | <1.0 |  |  |  |  | 1 |  |  |
| **Scrophulariaceae** | **Verbascum** |  | <1.0 | <1.0 | 4.5 | 10 | 0.6 | 1.8 | 1.3 | 3.2 | <1.0 | **9** | 2.4 | 3.2 |
|  | **%NL** | 2.9 | 2.5 | 11.5 | 33.2 | 38.7 | 38.3 | 20.2 | 20.4 | 8.79 | 29.9 |  | 20.6 | 13.9 |
|  | **number of taxa** | 15 | 16 | 23 | 19 | 22 | 24 | 28 | 39 | 16 | 33 |  | 24 | 8 |
|  | **PG / 10g honey** | 141658 | 175643 | 92400 | 104931 | 53711 | 157560 | 210258 | 134568 | 118532 | 84612 |  | 127387 | 46460 |
|  | **PG Nect. / 10g honey** | 137550 | 171252 | 81774 | 70094 | 32925 | 97215 | 167786 | 107116 | 108113 | 59313 |  | 103314 | 45310 |
|  | **HDE/P** | 0.03 | 0.01 | 0.01 | 0.01 | 0.10 | 0.12 | 0.15 | 0.15 | 0.01 | 0.90 |  | 0.15 | 0.27 |

**6. Supplementary Figure 7.** (**A**) Total Ion Chromatogram (TIC) and Selected Ion Monitoring (SIM) chromatograms of abscisic acid encountered isomers in an indicative Anama honey sample extract using HPLC-PDA-ESI/MS, (**B**) Total Ion Chromatogram (TIC) and Selected Ion Monitoring (SIM) chromatograms of (+)-abscisic acid solution in MeOH (at 2 μg/mL) using HPLC-PDA-ESI/MS

| **A** | **B** |
| --- | --- |
| 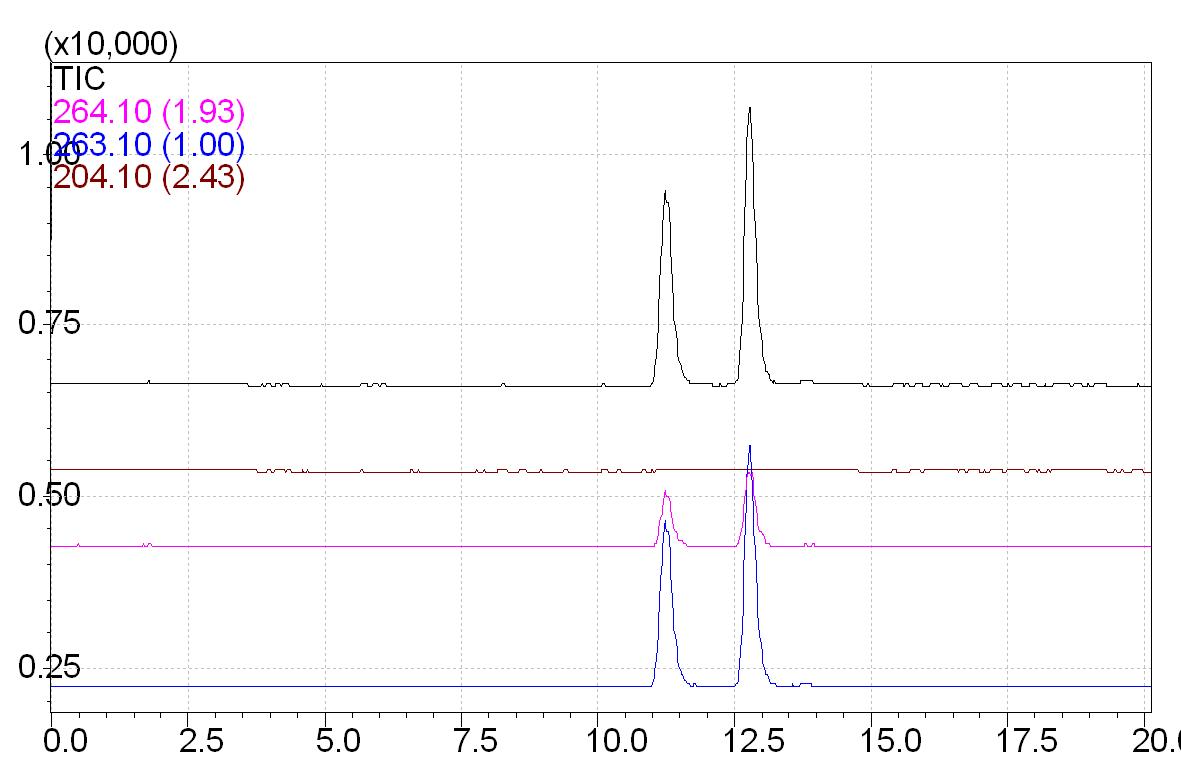 | 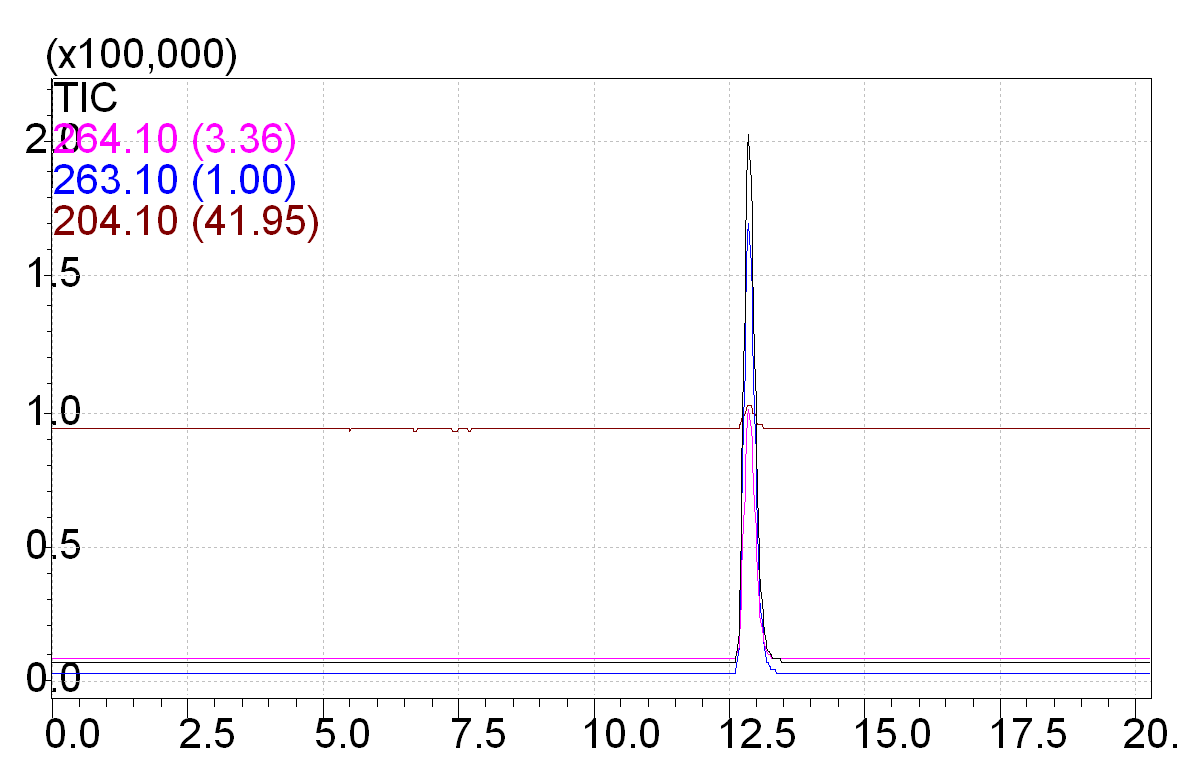 |

**7. UHPLC-HRMS quantitative analysis of abscisic acid**

*Ultrahigh Performance Liquid Chromatography Orbitrap High Resolution Mass Spectrometry Analysis of Honey Extracts*

The chromatographic conditions used for the quantitation of abscisic acid were identical to the ones reported in the main manuscript. Since the two isomers were not separated in the UHPLC-HRMS analysis quantitation considered their ratio after separation by HPLC-PDA-ESI/MS (see respective paragraph below, ratio of 2:3, Figure S1), using 204.1151 as quantitation ion. The calibration range for (+)-abscisic acid varied from 50-1000 ng/g _honey_ (equivalent to 50-1000 ng/mL), demonstrating an acceptable coefficient of determination, r^2^>0.9987. Standard addition was used for the recovery study (n=3) at two concentration levels (50 and 500 ng/g _honey_), exhibiting mean recoveries in the range of 81-92%. To facilitate the assessment of recovery of (+)-abscisic acid an organic citrus honey sample devoid of abscisic acid was utilized (in the fortification experiments).

**8. HPLC-PDA-ESI/MS separation of abscisic acid isomers**

*High Performance Liquid Chromatography Photo Diode Array -* *Electron Spray Ionization* *Mass Spectrometry (*HPLC-PDA-ESI/MS*)*

A Shimadzu (Kyoto, Japan) LCMS-2010 EV Liquid Chromatograph Mass Spectrometer instrument was used with the LCMS solution version 3.0 software consisting of an SIL-20A prominence autosampler and an SPD-M20A diode array detector (190-800 nm). The latter were coupled in series with a mass selective detector equipped with an atmospheric pressure ionization working in the negative Electron Spray Ionization (ESI-) mode. Detector voltage was set at 1.8 kV. The LC separation was achieved on a Fortis C18 column (2.1 x 150 mm, 3 μm, Fortis® Technologies Ltd., Neston, Cheshire, UK) thermostated at 30 °C, using a gradient system consisting of 0.1% formic acid in water (channel A) and acetonitrile (channel B). The flow rate was set at 0.3 mL min^-1^ and the column gradient program consisted initially of 20% B, ramped linearly over the course of 10 min to 30 % B. Afterwards, a linear increase to 35% B was implemented until the 20 min. Then, the mobile phase composition returned in the course of 2 min at 20% B. The mobile phase was held at 20%B for additional 3 min. The total runtime was 25 min. Identification of the analyte was achieved by comparing the retention time and UV absorption spectrum with those of the analytical standard. Confirmation was performed with mass spectrometry, functioning in the selected ion monitoring mode (SIM), monitoring, at least two ions (in total three ions, at 264.1, 263.1, and 204.1 m/z). The third diagnostic ion at 204.1 m/z was encountered reproducibly and more easily in elevated (>1 μg/mL) injected concentrations of (+)-abscisic acid.
